# Nature stays natural: two novel chemo-enzymatic one-pot cascades for the synthesis of fragrance and flavor aldehydes†

**DOI:** 10.1039/d3gc04191c

**Published:** 2023-12-22

**Authors:** Stefan Giparakis, Margit Winkler, Florian Rudroff

**Affiliations:** a TU Wien, Institute of Applied Synthetic Chemistry Getreidemarkt 9 163-OC 1060 Vienna Austria florian.rudroff@tuwien.ac.at; b TU-Graz, Institut für Molekulare Biotechnologie Petersgasse 14 8010 Graz Austria; c Austrian Center of Industrial Biotechnology (ACIB GmbH) Krenngasse 37 8010 Graz Austria

## Abstract

Novel synthetic strategies for the production of high-value chemicals based on the 12 principles of green chemistry are highly desired. Herein, we present a proof of concept for two novel chemo-enzymatic one-pot cascades allowing for the production of valuable fragrance and flavor aldehydes. We utilized renewable phenylpropenes, such as eugenol from cloves or estragole from estragon, as starting materials. For the first strategy, Pd-catalyzed isomerization of the allylic double bond and subsequent enzyme-mediated (aromatic dioxygenase, ADO) alkene cleavage were performed to obtain the desired aldehydes. In the second route, the double bond was oxidized to the corresponding ketone *via* a copper-free Wacker oxidation protocol followed by enzymatic Baeyer–Villiger oxidation (phenylacetone monooxygenase from *Thermobifida fusca*), esterase-mediated (esterase from *Pseudomonas fluorescens*, PfeI) hydrolysis and subsequent oxidation of the primary alcohol (alcohol dehydrogenase from *Pseudomonas putida*, AlkJ) to the respective aldehyde products. Eight different phenylpropene derivatives were subjected to these reaction sequences, allowing for the synthesis of seven aldehydes in up to 55% yield after 4 reaction steps (86% for each step).

## Abstract 2

We developed two novel chemo-enzymatic strategies to produce high-value fragrances and flavours derived from renewable phenylpropene-metabolites in up to 55% yield after four reaction steps.

## Graphical abstract

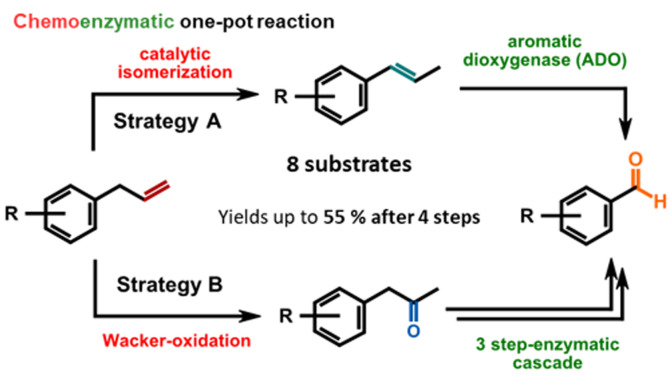

## Introduction

Growing concerns about the human impact on the environment prompt a reevaluation of the current chemical industry. Key chemicals vital for modern living, pharmaceutical precursors, and agrochemicals such as fertilizers are still derived from fossil fuels, contributing to unsustainability and environmental harm.^1^ Addressing these issues, Anastas and Warner introduced the twelve principles of green chemistry in 1998.^2^ These principles advocate for renewable resources, catalytic methods, and cascade-type transformations to create environmentally friendly alternatives. Inspired by nature's biosynthetic processes, chemists have developed chemo-enzymatic catalytic cascades using renewable materials.^3,4^ This approach enhances atom efficiency by capitalizing on (bio)catalysis, utilizing ambient conditions and water as a solvent.^5^ Integrating multiple steps minimizes waste and boosts energy efficiency, contributing to a more sustainable and eco-friendly synthesis. These considerations were the starting point for our research.

Herein, we present a proof of concept for two novel chemo-enzymatic sequential reaction cascades allowing for the production of aromatic aldehydes (*e.g.*, vanillin, piperonal, *p*-anisaldehyde, veratraldehyde and benzaldehyde). Aromatic aldehydes, vital for the food, fragrance, and chemical industries, face high demand beyond their natural availability.^6,7^

Vanillin, a major target molecule, is conventionally synthesized from guaiacol and glyoxylic acid under basic conditions, constituting 85% of annual production.^8,63^ Piperonal, anisaldehyde, and benzaldehyde emerge from diverse synthesis methods.^6,9–11^ Despite profitability, these processes are unfavorable due to their dependence on toxic reagents, hazardous materials, petrochemicals, and high energy demand.

Over the last few years, enzymatic processes have also gained relevance in the production of aldehydes, especially in the production of vanillin.^12–15^ Biological production of aldehydes offers a key advantage through its implementation of mild and comparably green reaction conditions. However, these *in vivo* enzymatic methods often suffer from moderate yields, a result of rapid interconversion to the corresponding alcohols or carboxylic acids. Some strategies to overcome this obstacle are utilizing improved reaction conditions, biphasic systems, or genetically modified strains with reduced aromatic aldehyde reduction propensity.^16^ Additionally, employing thermostable enzymes and conducting biotransformations at temperatures ≥50 °C also reduce by-product formation by deactivating endogenous enzymes.^17^

Nevertheless, even the most efficient and environmentally benign process is futile without a suitable starting material. Consequently, significant scientific efforts have been focused on utilizing renewable resources as starting materials for such synthetic methods. Vanillin, as already mentioned, a focal point in this type of research, has been successfully synthesized utilizing biotechnological means out of a plethora of different renewable resources, *e.g.*, ferulic acid, glucose, vinyl guaiacol, and (iso)eugenol.^22–29^ In particular, the conversion of isoeugenol proved quite promising, utilizing yeast (4.2 g L^−1^ vanillin with 52% conversion after 32 hours) or recombinant enzymes (225 mM vanillin obtained with a yield of 82.3%).^24,28^ Many methods are ultimately limited by high substrate cost (*e.g.*, ferulic acid), low titer concentrations (*e.g.*, glucose), product degradation, and the general complexity of these systems. Additionally, examples of the enzymatic production of other natural aromatic aldehydes from natural resources remain relatively sparse.^30–33^ A highly explored renewable material is lignin, which is obtained from wood after separation from cellulose and hemicellulose and consists of a polymeric network of mono-, di- or nonmethoxylated phenylpropanoids.^34^ Chemical methods for direct lignin-to-vanillin conversion have existed for a century but face challenges such as high energy consumption, low selectivity, and pollution.^8^ To effectively utilize lignin monomers, extensive pretreatment in the form of depolymerization of the biopolymer is necessary, which traditionally requires high temperatures, chemicals or catalysts.^35,36^ Modern developments in the field of lignin depolymerization are plentiful and a plethora of different reviews have summarized the technological advancement in the field over the last years.^35–39^ Recent examples include reductive catalytic fractionation (RCF), selectively liberating constituents like 4-alkylguaiacol, which can be biocatalytically converted into vanillin (18% yield based on RCF lignin oil) as described in recent articles.^40,41^ Novel investigations also explore biocatalyzed lignin degradation and valorization as a solution to these challenges.^35,42–46^ Certain fungi and bacteria, producing extracellular laccases or peroxygenases, selectively cleave lignin linkages.^47,48^ Unfortunately, the efficiency of enzymatic depolymerization remains low, hindering its application in lignin valorization.^36^ In summary, the overall complexity of lignin valorization and the limited product profile (mainly vanillin, syringaldehyde, and veratraldehyde) pose significant limitations.

**Fig. 1:**
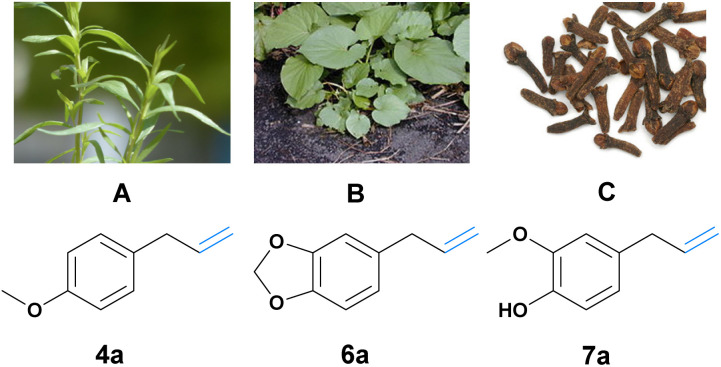
(A) Estragole 4a can be isolated from estragon.^18^ (B) Safrole 6a can be isolated from makulan.^19^ Picture: Forest & Kim Starr (CC BY 3.0). (C) Eugenol 7a can be isolated from cloves.^20,21^ The image was adapted from Brian Arthur (CC BY-SA 4.0).

In this study, the focus shifts to exploring phenylpropenes, such as estragole (4a), safrole (6a), and eugenol (7a), as renewable candidates for the synthesis of high-value aldehydes (Fig. 1). In contrast to lignin that, as mentioned above, needs extensive pretreatment, phenylpropenes are readily available and easily isolated from natural resources through hydro or steam distillation (see Fig. 1). Phenylpropenes also offer a chemical advantage due to their shared structural motif with naturally occurring aromatic aldehydes such as eugenol (vanillin), safrole (piperonal), and estragole (anisaldehyde). A system accommodating various phenylpropene constituents would enable the synthesis of a plethora of different aromatic aldehydes, overcoming the product limitations of other systems mentioned earlier. Their preinstalled allylic functionality is also easily functionalized. Currently, the use of some phenylpropenes (*e.g.*, eugenol, estragon, and veratraldehyde) is mainly limited to the flavor and fragrance industries, and also, to some extent, to the chemical and enzymatic synthesis of aromatic aldehydes.^6^ Additionally, naturally isolated phenylpropenes are also comparably cheap (*e.g.*, natural safrole, eugenol and estragon can all be obtained for around 20–40 € per kg), even to the respective synthetically produced aldehydes, of course disregarding the additional negative environmental impact of their synthesis. In summary, phenylpropenes are an ideal and underused natural resource for the biological and environmentally benign synthesis of aromatic aldehydes.

**Scheme 1:**
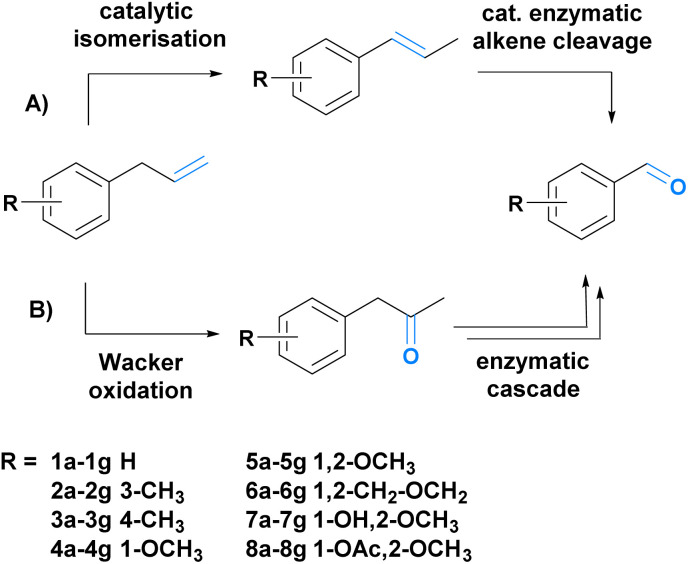
Two novel chemo-enzymatic sequential one-pot reactions developed in this work. (A) Consists of a two-step cascade based on a Pd-assisted double bond isomerization and a subsequent enzymatic C=C double bond cleavage. (B) Cascade B starts with the double bond oxidation to the corresponding ketone and is followed by an enzymatic cascade to yield the desired fragrance and flavour aldehydes.

To achieve our goal, we envisioned two different synthetic strategies (Scheme 1A and B). The first route comprises the catalytic isomerization of the allylic double bond followed by enzymatic alkene cleavage (Scheme 1A).^49,50^ In the second route, we combined a Wacker oxidation with three enzymatic steps (Baeyer–Villiger monooxygenation, ester cleavage, and alcohol oxidation).^51–53^ The Wacker oxidation leads to the ketone functionality which is oxidized to the corresponding ester. Subsequent esterase-mediated hydrolysis results in the primary alcohol, which is further oxidized to the desired aldehyde (B, Scheme 1). To demonstrate the scope of the transformations, eight substrates (Scheme 1) (1a–8a) were chosen, five of which can be isolated from nature (5a–8a).^6^ Both cascades were investigated and compared.

## Results

**Scheme 2:**
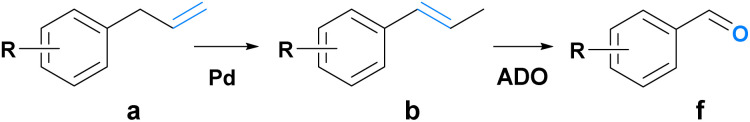
Route A. Compound a is isomerized to b and subsequently oxidized to the corresponding aldehyde f.

Route A consists of two steps (Scheme 2). First, a chemical isomerization converts the allylic double bond of the phenylpropene into the vinylic counterpart. For this, a solvent-free palladium(ii) chloride (PdCl_2_)-catalyzed isomerization procedure under neat conditions was investigated. It allowed for the selective isomerization of all substrates 1a–8a with almost exclusive formation of the respective *E*- isomer (1b–8b, determined from ^1^H NMR, ESI chapter 6.2†) and quantitative conversions for all, except 8b (Table 1). The catalyst could additionally be isolated and reused, as confirmed by testing in three consecutive isomerizations following the initial reaction (ESI chapter 5.18†).

**Table 1:** Results of the solvent-free PdCl_2_-catalyzed isomerization of phenylpropenes 1b–8b

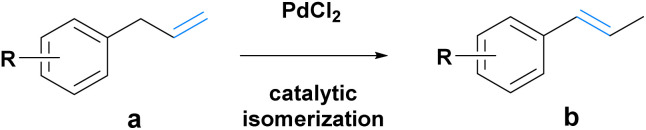			
Substrate^a^	Cat.	Temp.	Conversion
1a	5.0 mol%	rt	Quant.
2a	5.0 mol%	rt	Quant.
3a	5.0 mol%	rt	Quant.
4a	5.0 mol%	40 °C	Quant.
5a	5.0 mol%	40 °C	Quant.
6a	5.0 mol%	40 °C	Quant.
7a	5.0 mol%	rt	Quant.
	2.5 mol%	rt	Quant.
8a	5.0 mol%	40 °C	94%

a Conditions: 100 mg substrate (0.48–0.8 mmol), catalyst PdCl_2_ (2.5–5 mol%), neat conditions, rt to 40 °C, 24 h.

Following the isomerization, the obtained reaction mixture was directly employed in the subsequent biotransformation after being diluted with ethanol (EtOH) to a 0.5 M concentration. The aromatic dioxygenase (ADO) identified by Ni *et al.* was chosen for the enzymatic conversion due to its notable characteristics.^29^ This choice was based on the enzyme's ability to conduct transformations without requiring a cofactor or metabolic redox equivalents. Furthermore, the enzyme exhibited exceptional substrate promiscuity, as demonstrated with various substrates such as 4-vinyl guaiacol, isoeugenol 7b, and anethol 4b. Protein production was performed in *Escherichia coli* [*E. coli*., BL21 (DE 3)] transformed with a pET21 vector entailing the gene of interest, together with an ampicillin resistance gene. Protein expression was controlled using an isopropyl β-d-1-thiogalactopyranoside (IPTG) inducible promoter. Resting cells were obtained after harvesting and washing the main culture, and the biotransformation was then performed *via* whole-cell biocatalysis (OD_590_ 30, 5–8 mM substrate concentration, in 50 mM phosphate buffer, pH 7.4) at 50 °C. This temperature was chosen firstly because ADO has the highest reported activity at this temperature, and secondly to prevent the formation of the corresponding alcohol through the thermal deactivation of native *E. coli* alcohol dehydrogenases (ADHs). The isomers of all eight phenylpropenes were subjected to the enzyme in 1 ml scale experiments. Samples for gas chromatography (GC) analysis were taken after 0, 1, 5, and 24 hours. In contrast to the reported literature data, only 7b and 8b were successfully oxidized and (after hydrolysis of the phenolic acetate in 8b) were converted into vanillin 7f (ESI chapter 5.7†) with yields of 53% and 61%, respectively, after 24 hours.

It was hypothesized that the high substrate specificity in ADO stems from the dependence on hydrogen bonding from the hydroxy group at the *para* position of the aromatic moiety (as can be found in 7b) for substrate binding in the active site of the enzyme.

Mechanistic studies for a comparable dioxygenase (stilbene cleavage oxygenase from *Novosphingobium aromaticivorans*) suggested that a similar hydrogen bonding facilitated binding and activation of the olefinic bond.^54^ To test this hypothesis, anethol 4b was demethylated chemically and subjected to ADO in a large batch experiment (30 ml scale). After 24 hours, the reaction mixture was extracted with ethyl acetate, the solvent was removed, and the residue was analyzed by ^1^H NMR. Confirming the hypothesis, 4-(prop-2-en-1-yl)phenol was successfully converted into the respective 4-hydroxybenzaldehyde with a conversion of 12% (ESI Fig. 2†).

**Scheme 3:**
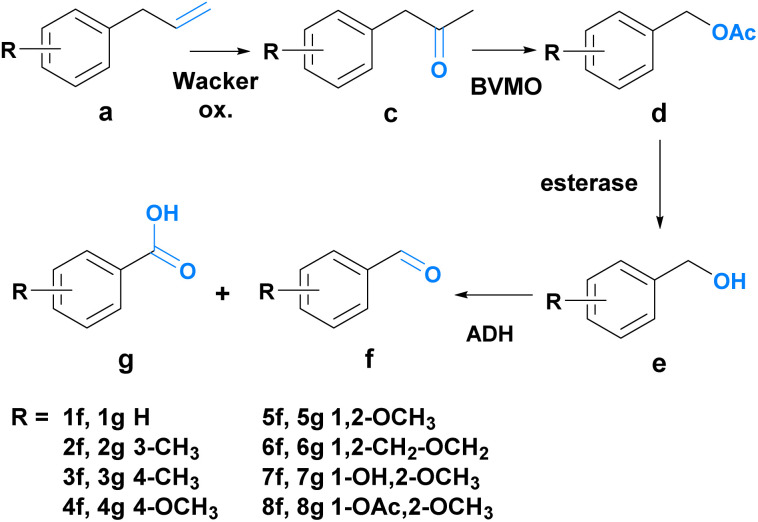
Overview of route B. Compound a is oxidized twice to c and then d, respectively. Ester cleavage affords e, which is again oxidized to f.

Since route A allowed only for the synthesis of one of the desired aldehydes (7f), route B was explored as an alternative to fill the product scope gap. Route B consists of four steps coupled into a sequential chemo-enzymatic reaction cascade (Scheme 3). The first step of the reaction sequence – the chemical transformation – involves a Wacker oxidation which converts phenylpropenes 1a–8a into the corresponding phenylacetones 1c–8c. An enzymatic Baeyer–Villiger oxidation leads to the formation of the acetate esters 1d–8d, which are then enzymatically hydrolyzed to liberate the aromatic alcohols 1e–8e. After the final enzymatic oxidation, the aldehydes 1f–8f are obtained. For the Wacker oxidation, a method developed by González-Martínez *et al.* was adapted since it seemed most promising for achieving biocompatibility for our system.^55^

It uses palladium(ii) trifluoroacetate [Pd(TFA)_2_] and sodium trifluoroacetate (NaTFA) as the catalytic system and iron(iii) chloride (FeCl_3_) (or iron(iii) sulfate [Fe_2_(SO_4_)_3_] for 2a and 3a) as the terminal oxidant, necessary for the reoxidation of the metal catalyst. The reaction was performed in water with 5% acetonitrile (ACN) as a co-solvent. For the Baeyer–Villiger oxidation, the literature known Baeyer–Villiger-monooxygenases (BVMOs), namely phenylacetone monooxygenase (PAMO) from *Thermobifida fusca* and cyclohexanone monooxygenase (TmCHMO) from *Thermocrispum municipale* were utilized.^56–58^ Protein production was carried out in *E. coli* (TOP10) transformed with a pBAD vector entailing the gene of interest, together with an ampicillin resistance gene. Protein expression was controlled using an l-arabinose inducible promoter. In the third stage of the reaction sequence, the hydrolysis of the generated acetate ester was facilitated by an esterase from *Pseudomonas fluorescens* (PfeI). This enzyme was employed in the form of a lyophilized cell powder. For the last step, the literature-known, membrane-bound, flavin adenine dinucleotide (FAD)-dependent alcohol dehydrogenase AlkJ from *Pseudomonas putida* was selected.^59,60^ Also in this case, protein production was performed in *E. coli* (BL21 DE3) transformed with a pkA1 vector entailing the gene of interest and a chloramphenicol resistance gene. The enzyme expression was controlled using an IPTG inducible promoter. To assess the appropriateness of our chosen enzymes, initial biotransformations were carried out again on a 1 ml scale using the respective whole-cell biocatalyst (5 mM substrate concentration, OD_590_ 20, in 50 mM phosphate buffer, pH 7.4) or the lyophilized powder. The following substrate enzyme combinations were tested: 1c–8c with PAMO and TmCHMO, 1e–8e with AlkJ and 1d with PfeI. The samples of the biotransformation (150 μl) were taken after 0, 1, 5, and 24 hours and subjected to GC analysis to monitor product formation. All enzymes were able to convert the respective substrate into the desired product, except for TmCHMO in the case of 7a and 8a (ESI Fig. 3–5†).

After the successful validation of the substrate promiscuity of our enzymes, the enzymatic cascade was assembled. A mixed-culture methodology was employed to eliminate the need for an external cofactor recycling system and to ensure utmost simplicity. In this approach, enzymes were individually produced in distinct host cell cultures. Following the harvesting and washing of cells after 20 hours of protein production, they were combined, along with the lyophilized PfeI powder, to create the consolidated whole-cell biocatalyst (OD_590_ 20 for AlkJ and the corresponding BMVO, 150 U PfeI, in 50mM phosphate buffer, pH 7.4). To validate the concept, the enzymatic cascade was assessed using purified substrates 1b–8b (5 mM) on a 1 ml scale and a time-dependent experiment (*T* = 0, 1, 5, and 24 hours). All eight tested substrates were effectively transformed into their respective aldehydes, barring substrate 8a, which produced 7f due to the hydrolysis of the phenolic acetate. The yields varied across the substrates, ranging from 11% for 1a to 41% for 4a (only conducted with PAMO, ESI Fig. 6†). Besides the desired products in the form of aromatic aldehydes 1f–7f, major overoxidation to the respective carboxylic acids 1g–7g over a course of 24 hours was also encountered. Nevertheless, complete conversion of the phenylpropanones was almost always achieved, which was a promising sign for the viability of our system.

In the course of the reaction, we detected a significant loss in material, which we summarized as “not recovered”. A closer look revealed that the missing material was the carboxylic acid. The “not recovered” label depicts the difference between the theoretical mass balance and the observed mass recovery (sum of each component, ESI Fig. 6†).

**Fig. 2:**
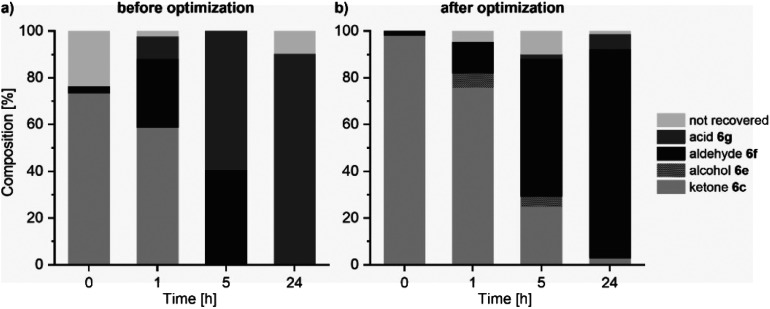
Optimization of the chemo-enzymatic cascade (route B) to avoid the production of the unwanted side product, the carboxylic acid. (a) Non-optimized conditions. (b) Optimized conditions – see the text. The “not recovered” label represents the mass difference between the theoretical mass balance and the observed mass recovery.

Lastly, the whole chemo-enzymatic sequential reaction cascade was performed (ESI Fig. 7 for PAMO and Fig. 8† for TmCHMO). For this, the reaction mixture of the Wacker oxidation (20 vol% of the biotransformation mixture) was used directly without any purification or isolation for the subsequent biotransformation using the mixed culture biocatalyst, as described above. Adjusting the pH of the reaction mixture after performing the Wacker oxidation by adding 2 M sodium hydroxide solution or just by adding the buffered cell suspension leads to the precipitation of iron oxides and the catalyst. This allows for the removal of the reagents from the first step by centrifugation. Interestingly, we also discovered that leaving the solids in the reaction mixture had no observable adverse effect on the biocatalytic system. Again, overoxidation of the formed aldehydes to the respective carboxylic acids was observed. Additional optimization of the system was necessary to improve aldehyde accumulation after 24 hours (Fig. 2). One clear goal on the way to high aldehyde titers was the suppression of overoxidation events (Fig. 2a). For the quantification of the carboxylic acid, the analytic system was changed from GC to high-pressure liquid chromatography (HPLC) analysis. Through the addition of 2.5 vol% EtOH to the biotransformation mixture and by increasing the molarity of the phosphate buffer from 50 mM to 150 mM, the amount of carboxylic acid was reduced by roughly 70% for substrate 6c (ESI Fig. 9†). The increased buffer concentration balanced the pH value over the course of the reaction. The addition of EtOH led to a reduced ADH activity toward the actual substrate through inhibition which also had a positive effect on the aldehyde formation.

For the optimized conditions, PAMO was chosen as the BVMO since it showed similar characteristics to TmCHMO but with a broader substrate scope. Aldehydes were obtained in moderate to good yields (7% to 55%) after four steps, corresponding to a theoretical step yield of 50% for 7f and 86% for 6f (Table 2 and ESI Fig. 10†). The step yield corresponds to the theoretical yield for each step, which would have been necessary for a conventional synthetic step-by-step approach to achieve the same results.

**Table 2:** Yields of the chemo-enzymatic one-pot reaction cascade for PAMO *via* route B

Entry^a^	Yield of Wacker Ox.^b^	Yield of enzymatic cascade^c^, *t* 24 h	Yield combined	Ratio of aldehyde/acid
1a	75%	53 ± 11% (80%)^d^	38 ± 7% (78%)	2.7/1
2a	63%	61 ± 22% (83%)	38 ± 13% (78%)	2.9/1
3a	51%	86 ± 11% (95%)	43 ± 8% (81%)	6.2/1
4a	31%	62 ± 19% (84%)	20 ± 5% (66%)	11.1/1
5a	50%	58 ± 7% (83%)	29 ± 5% (72%)	16.6/1
6a	62%	89 ± 7% (96%)	55 ± 8% (86%)	14.2/1
7a	33%	20 ± 10% (56%)	7 ± 4% (50%)	500/1
8a^e^	43%	16 ± 4 (55%)	7 ± 2% (50%)	—

a Conditions: 1 ml reaction volume, OD_590_ 20 for PAMO and AlkJ as thawed whole cells, 2.5 vol% EtOH, 150 U PfeI (lyophilized powder), 100–150 mM PBS (pH 7.4), no removal of the iron precipitate, no further pH adjustment, 20 vol% Wacker mixture (∼1.3–4 mM), 33 °C, 220 rpm, 24 h, all reactions were performed in triplicate.

b Yield determined by (U)HPLC at the moment of the addition of the biocatalyst.

c Yield determined by (U)HPLC analysis.

d Number in the bracket corresponds to the theoretical yield for each individual step in the cascade *e.g.*, step 1, 2, 3, or 4.

e Aldehyde 7f was obtained as the product of the transformation.

## Discussion

We explored two novel cascade reactions for the valorization of natural phenylpropenes for the synthesis of valuable aromatic aldehydes (and their corresponding carboxylic acids).

Route A gave only one whereas route B afforded all of the desired aldehydes in moderate to good yields. More importantly, it was possible to perform all reaction sequences in a one-pot fashion without necessary derivatization or separation while employing only catalytic or enzymatic steps. The desired starting material in the form of natural phenylpropenes proved to be a viable choice for this conversion. All reactions were performed under almost neat conditions in water with low amounts of organic co-solvents (*e.g.*, 5% ACN). For route A, we investigated PdCl_2_-mediated isomerization under neat conditions at room temperature, which even allowed for the recovery of the metal catalyst. This remarkable efficiency was only dulled by the poor substrate acceptance of ADO, which allowed only for the production of vanillin 7f. Although this unoptimized route may not achieve the highest yield compared to other established methods for the production of vanillin from isoeugenol (as described in the Introduction) (*e.g.*, ∼4 mM here *vs.* 225 mM), the isomerization procedure might help to open the door for the efficient utilization of other enzyme classes that catalyze the enzymatic cleavage of 1-propenylbenzenes for the synthesis of aromatic aldehydes.^33^ We already discovered a novel dioxygenase capable of transforming up to 50 mM isoeugenol within 5 h into the corresponding vanillin (data not shown). Additionally, work is currently underway in our group to increase the substrate profile of ADO by rational protein design.

Route B, however, allowed for the synthesis of all desired aldehydes. Of course, the necessary terminal oxidant for the Wacker oxidation in the form of an Fe(iii) salt makes this step catalytic only with respect to the metal catalyst. Employing a different procedure for the Wacker oxidation of phenylpropenes that relies on a green terminal oxidant, such as oxygen, for the reoxidation of the metal catalyst proved difficult due to the tendency of catalyst decomposition (*i.e.*, palladium black) and side product formation. However, compared to the traditional conditions for the Wacker oxidation, which employ hazardous copper salts as the terminal oxidant, Fe(iii) salts can be seen as “environmentally benign” alternatives. The scarcity issue, which plagues the stoichiometric use of other transition metals, is also not an issue since iron is highly abundant in nature. Biocatalytically speaking, the method suffers from low yields for substrates 7a and 8a due to the poor substrate acceptance of the BVMOs PAMO and especially TmCHMO, the latter yielding no product. Protein engineering would be necessary to increase the substrate scope and activities of the BVMOs. The remaining substrates (1a–6a) were transformed into the desired aldehydes with good selectivity, and moderate yields were obtained after 4 steps. Step yields, however, were excellent. This was achieved by improving the biotransformation conditions through the addition of EtOH and adjusting the buffer concentration, avoiding the formation of the undesired carboxylic acids almost entirely. In the case of the optimized reaction parameters, the main reason for diminished yields in the enzymatic cascade is the incomplete conversion of the intermediates (ketone or alcohol) and is not mainly a result of by-product formation. This limitation is imposed by enzyme inhibition through the addition of EtOH. In comparison with other literature-known procedures for the enzymatic synthesis of aromatic aldehydes, the strength of route B lies in its impressive product scope. Additionally, since the enzymatic cascade is assembled modularly, removing one or more enzymes from the cascade would allow for the production of the intermediates (*e.g.*, alcohol or acetate) of the synthetic route instead, further expanding the scope of accessible products. Aromatic alcohols and their corresponding acetates (*e.g.*, benzyl alcohol and benzyl acetate) are important fragrance compounds and are used extensively in the fragrance and perfume industry.^6,61,62^ Optimization of the 4-step cascade is ongoing, as well as the identification of novel alkene cleaving enzymes to broaden the scope of the presented cascade. In summary, route A allowed for the synthesis of vanillin from eugenol and eugenol acetate whereas route B allowed for the efficient transformation of all other tested phenylpropenes into the corresponding aromatic aldehydes.

## Author contributions

F.R. and M.W. led the project, conceived the research, and designed the experiments. S.G. performed the lab work and the synthesis of the starting materials and reference compounds. He also expressed and screened the enzymes. S.G., M.W., and F.R. co-wrote the manuscript and designed the figures. All authors commented on the manuscript.

## Conflicts of interest

There are no conflicts to declare.

## Supplementary Material

GC-026-D3GC04191C-s001
