# Supplementary material for: Nature stays natural: two novel chemo-enzymatic one-pot cascades for the synthesis of fragrance and flavor aldehydes
Source: Green Chem. 2023 Dec 22;26(3):1338–44. doi: 10.1039/d3gc04191c (PMC10840651; doi:10.1039/d3gc04191c)
Supplement: GC-026-D3GC04191C-s001 [file GC-026-D3GC04191C-s001.pdf]

Electronic Supplementary Material (ESI) for Green Chemistry.
This journal is © The Royal Society of Chemistry 2023

Supporting Information

Nature stays natural: Two novel chemo-enzymatic one-pot cascades for the synthesis of fragrance & flavor aldehydes

Stefan Giparakis,[1] Margit Winkler,[2,3] Florian Rudroff*,[1]

[1]TU Wien, Institute of Applied Synthetic Chemistry, Getreidemarkt 9, 163-OC, 1060 Vienna, Austria

[2]TU Graz, Institut für Molekulare Biotechnologie, Petersgasse 14, 8010 Graz, Austria

[3]Austrian Center of Industrial Biotechnology (ACIB GmbH), Krenngasse 37, 8010 Graz, Austria

Contents

1. General remarks ... 3

2. Sample preparation for GC analysis ... 3

3. Sample preparation for HPLC analysis ... 3

4. GC and HPLC analysis and methods ... 4

4.1 GC-Analysis ... 4

4.2 (U)HPLC-Analysis ... 4

5. General Procedures ... 5

5.1 General procedure for protein production and biotransformation with whole-cell biocatalysts ... 5

5.2 Gene expression and protein production conditions for ADO, PAMO, TmCHMO, and AlkJ 6

5.3 Enzymatic cascade using a whole-cell mixed culture biocatalyst with PAMO/TmCHMO, AlkJ and PfeI ... 6

5.4 Chemo-enzymatic one-pot reaction using a whole-cell mixed culture biocatalyst with PAMO /TmCHMO, AlkJ, and PfeI ... 7

5.5 Optimized procedure for the chemo-enzymatic one-pot reaction using a whole-cell mixed culture biocatalyst with PAMO /TmCHMO, AlkJ, and PfeI ... 7

5.6 Preparation of lyophilized cells containing PfeI ... 7

5.7 ADO chemo-enzymatic one-pot biotransformation and substrate scope ... 8

5.8 ADO biotransformation with 4-(prop-2-en-1-yl)phenol ... 8

5.9 AlkJ, PAMO, and TmCHMO substrate scope ... 9

5.10 PAMO, PfeI and AlkJ enzymatic cascade with purified substrate -GC analysis ... 11

5.11 PAMO, PfeI and AlkJ chemo-enzymatic cascade -GC analysis ... 12

5.12 TmCHMO, PfeI and AlkJ chemo-enzymatic cascade -GC analysis ... 13

5.13 Optimization of the chemo-enzymatic sequential reaction cascade –(U)HPLC analysis ... 14

5.14 PAMO, PfeI, and AlkJ chemo-enzymatic cascade –(U)HPLC analysis ... 15

5.15 Large-scale biotransformation using 4a ... 16

5.16 General procedure for the Wacker-oxidation of phenylpropenes[2] 17
5.17 General procedure for the isomerization of phenylpropenes 17
5.18 Isomerization and catalyst recovery 18
6. Synthesis 18
6.1 Synthesis of 4-(prop-2-en-1-yl)phenol[3] 18
6.2 Isomerization 19
6.2.1 Synthesis of (*E*/*Z*)-1-(prop-1-en-1-yl)benzene 1b 19
5.2.2. Synthesis of *(E/Z)*-1-methyl-3-(prop-1-en-1-yl)benzene 2b 20
5.2.3. Synthesis of *(E/Z)*-1-methyl-4-(prop-1-en-1-yl)benzene 3b 20
5.2.4. Synthesis of *(E/Z)*-1-methoxy-4-(prop-1-en-1-yl)benzene 4b 20
5.2.5. Synthesis of (*E*/*Z*)-1,2-dimethoxy-4-(prop-1-en-1-yl)benzene 5b 20
5.2.6. Synthesis of (*E*/*Z*)-5-(prop-1-en-1-yl)-2*H*-1,3-benzodioxole 6b 21
5.2.7. Synthesis of (*E*/*Z*)-2-methoxy-4-(prop-1-en-1-yl)phenol 7b 21
5.2.8. Synthesis of (*E*/*Z*)-2-methoxy-4-(prop-1-en-1-yl)phenol acetate 8b 21
6.3 Wacker-Oxidation 21
6.3.1 Synthesis of phenylpropan-2-one 1c 21
5.3.2. Synthesis of 1-(3-methylphenyl)propan-2-one 2c 22
5.3.3. Synthesis of 1-(4-methylphenyl)propan-2-one 3c 22
5.3.4. Synthesis of 1-(4-methoxylphenyl)propan-2-one 4c 22
5.3.5. Synthesis of 1-(3,4-dimethoxylphenyl)propan-2-one 5c 22
5.3.6. Synthesis of 1-(2H-1,3-benzodioxol-5-yl)propan-2-one 6c 23
5.3.7. Synthesis of 1-(4-hydroxy-3-methoxyphenyl)propan-2-one 7c 23
5.3.8. Synthesis of 1-(4-acetoxy-3-methoxyphenyl)propan-2-one 8b 23
7. Appendix – NMR spectra 24
8. Gene Sequence 33
9. Literature 35

1. General remarks

If not mentioned otherwise, all glassware and media used for the cultivation of *E. coli* were sterilized via autoclavation prior use. All stock solutions of substrates used for the biotransformation were prepared in ethanol (EtOH) or Acetonitrile (ACN) (usually 0.5 M) and stored in the freezer at -20 °C for a limited amount of time. All stock solutions used for the cultivation of Escherichia coli (*E. coli)* and the induction of protein production (e.g., antibiotics, inducer) were prepared in water or EtOH and sterilized *via* sterile filtration through the use of a syringe filter. All substances used for the biotransformation or cultivation of *E. coli* and synthetic procedures were purchased from Sigma Aldrich or a comparable vendor for chemicals. The following practices were carried out in all synthetic procedures unless stated otherwise. All glassware was flame dried before use, and to guarantee water and oxygen exclusion for sensitive reactions, Schlenk techniques were employed. In general, reactions were carried out under slight argon pressure and stirred magnetically. Liquid reagents were added by syringe through a rubber septum, and solid reagents were added in a slight argon countercurrent.

NMR-analysis (^{1}H-NMR) was performed on the Bruker Avance 400 at 400 MHz. Depending on the substance, measurements were performed in chloroform-d6.

TLC analysis was performed using silica-gel-coated aluminum plates (Silica gel 60 F254, Merck). Anisaldehyde, basic $KMnO_4$, and phosphomolybdic acid/cerium sulfate solution were used as dip reagents to visualize the compounds.

The following solvents were used for synthesis or purification:

- ACN and DMSO were obtained in *p.a.* grade and used as received.
- H_2O was obtained from an ultrapure filtration system.
- PE and EtOAc used for column chromatography were obtained in technical grade.

2. Sample preparation for GC analysis

The aqueous sample taken from the biotransformation was transferred into a 1.5 ml Eppendorf vessel charged with 300 µl of ethyl acetate containing 1 mM of methyl benzoate as internal standard. The tube was shaken and centrifuged for 1 min at 14000 rpm. The organic phase was then transferred into a second Eppendorf vessel charged with Na_2SO_4. After a second centrifugation step for 1 min at 14000 rpm the supernatant was transferred into a GC vial and measured immediately or stored in the freezer at -30 °C for later measurement.

3. Sample preparation for HPLC analysis

The aqueous sample taken from the biotransformation was diluted with 100 µl of ACN in a 1.5 ml Eppendorf vessel. The mixture was centrifuged for 1 min at 14000 rpm and then transferred into an

HPLC vial *via* a syringe, passing the solution through a filter. Samples were then directly measured or stored in the freezer at -30 °C for later measurements.

4. GC and HPLC analysis and methods

4.1 GC-Analysis

GC analysis for the extracted samples, as described in chapter **2**, was performed on a Thermo Fisher Scientific Trace 1310 Dual GC composed of an FID detector and two Rxi-5Sil MS columns (length 15, ID 0.25 mm, film thickness 1.0 µm). GC quantification was performed with internal referencing employing 1 mM of methyl benzoate as an internal standard.

Method: Carrier-gas: He; Injection volume: 1 µl (split); Injector: 280 °C; Gas-flow: 1.5 ml/min, Temp.: 80 °C for 1 min, 80 -300 °C (60 °C/min), 300 °C for 4 min.

4.2 (U)HPLC-Analysis

HPLC analysis for the aqueous samples, as described in chapter **3**, was performed on either:

A) JASCO HPLC 2000Plus system: PU-2089 quaternary gradient pump; AS-2057 autosampler; CO-2060 column oven; LC-NetII ADC. Detector: MD-2018 PDA. Column: Agilent ZORBAX Eclipse XDB-C18, 5 µm, 4.6 x 150 mm.

Methods:

For all compounds in the same substrate class **1-8** (ketone, alcohol, aldehyde, and acid) the same analytical method was utilized.

1, 3, 4, 5: Solvent system: H_2O(0.1% FA) / ACN, Injection volume: 5 µl, Flow rate: 2 ml/ min, Column oven temperature: 25°C, 40 % ACN for 10 min.
2: Solvent system: H_2O (0.1% FA) / ACN, Injection volume: 10 µl, Flow rate: 2 ml/ min, Column oven temperature: 25°C, 20 % ACN for 10 min.

6: Solvent system: H_2O (0.1% FA) / ACN, Injection volume: 10 µl, Flow: 2 ml/min, Column oven temperature: 25°C, 30 % ACN for 10 min.

7, 8: Solvent system: H_2O (0.1% FA) / ACN, Injection volume: 5 µl, Flow rate: 2ml/min, Column oven temperature: 25°C, 5 % ACN for 4 min, gradient to 60 % ACN in 13 min, gradient to 5 % ACN in 2 min (19 min total).

B) Shimadzu Nexera X2 UHPLC system: Two LC-30AD solvent delivery units; SIL-30AC autosampler; DGU-20A3 solvent degasser; CBM-20A communications bus module; CTO-20AC

column oven; SIL-30AC autosampler. Detector: SPD-M20A PDA. Column: Waters XSelect CSH C18, 2.5 µm, 3.0 x 50 mm.

Methods:

For all compounds in the same substrate class **1-8** (ketone, alcohol, aldehyde, and acid), the same analytical method was utilized.

1-6: Solvent system: H_2O (0.1% FA)/ACN, Injection volume: 2.5 µl, Flow rate: 1.7 ml/min, Column oven temperature 40°C, 25 % ACN for 0.15 min, gradient to 80 % ACN in 2.05 min, 80 % ACN for 0.3 min, gradient to 25 % ACN in 0.01 min, 25% ACN for 0.490 min (3 min total).

7,8: Solvent system: H_2O (0.1% FA)/ACN, Injection volume: 2.5 µl, Flow rate: 1.7 ml/min, Column oven temperature 40°C, 5 % ACN for 0.15 min, gradient to 95 % ACN in 2.05 min, 95 % ACN for 0.3 min, gradient to 5 % ACN in 0.01 min, 5% ACN for 0.490 min (3 min total).

5. General Procedures

5.1 General procedure for protein production and biotransformation with whole-cell biocatalysts

A pre-culture containing the expression plasmid for the desired enzyme was grown in a 15 ml falcon tube charged with 5 ml LB-Miller medium, supplemented with the appropriate antibiotic for 19 hours at 37 °C and 220 rpm. The following day, a 20-500 ml flask was charged with TB-Medium (1/5 of the flask volume) and the appropriate antibiotic and inoculated with 1 % (v/v) of the pre-culture. The flask was then shaken at (37 °C, 150 – 170 rpm) until the culture reached an OD_{590} of 0.6. Subsequently, the appropriate inducer (and auxiliary salts) was added to the suspension. After shaking the culture for 22 hours at 20-25 °C at 150 rpm, the cells were harvested by centrifugation (6000 rcf, 10 min). To prepare the biocatalyst, the supernatant was removed, and the cell pellet was resuspended in sodium phosphate buffer (50 mM, pH 7.4). After a second centrifugation and removal of the supernatant, the cell pellet was resuspended in sodium phosphate buffer (volume determined by the desired OD) to obtain the washed biocatalyst. Biotransformation was performed in 8 ml screw cap vials. For this, 1 ml of the whole-cell biocatalyst was combined with the desired substrate dissolved in a co-solvent. Biotransformation was then performed at 30- 50 °C for 24 hours. Samples (150 µl or 100 µl) were taken after 0, 1, 5, and 24 hours and analyzed *via* GC or (U)HPLC.

5.2 Gene expression and protein production conditions for ADO, PAMO, TmCHMO, and AlkJ

Table 1: Typical conditions for gene expression and protein production for the enzymes used in this work.

Enzyme	Inducer	Expression temperature	Antibiotic	Biotransformation temperature	Auxiliary
ADO	IPTG (1 mM)	20-25 °C	Ampicillin	50 °C	$FeCl_2$ (1 mM)
PAMO	20 % L-Arabinose (2% v/v)	25 °C	Ampicillin	37 °C	-
TmCHMO	20 % L-Arabinose (2% v/v)	25 °C	Ampicillin	30 °C	-
AlkJ	IPTG (1mM)	25 °C	Chloramphenicol	30 °C	-

5.3 Enzymatic cascade using a whole-cell mixed culture biocatalyst with PAMO/TmCHMO, AlkJ and PfeI

A pre-culture of the respective *E. coli* strains containing the expression plasmid for the Baeyer-Villiger-monooxygenase (PAMO or TmCHMO), and the alcohol dehydrogenase (AlkJ) were grown in a 15 ml flacon tube filled with 5 ml of LB-Miller medium, supplemented with the appropriate antibiotic (1 mg / ml chloramphenicol for AlkJ and 1 mg / ml Ampicillin for PAMO) for 19 hours at 220 rpm. Subsequently, a 250 ml flask charged with TB medium and the appropriate antibiotic was inoculated with the respective pre-culture (one for the BVMO and one for the ADH). The cultures were grown until an OD_{590} of 0.5-0.6 was reached at 37 °C and 150-170 rpm. A stock solution of an appropriate inducer was then added to reach a final concentration of 0.2 vol% of 20 % L-Arabinose for PAMO or TmCHMO and 1 mM IMPT for AlkJ. The flasks were shaken at 150 rpm at 25 °C for 22 hours to produce sufficient protein. The next day the cells were harvested by centrifugation (6000 rfc, 10 min, 4 °C), washed with sodium phosphate buffer, and centrifuged again (6000 rcf, 10 min, 4 °C). Lastly, the cell pellet was resuspended with an appropriate volume of sodium phosphate buffer (50 mM, pH 7.4) to obtain a final OD of 40. The two cell suspensions were mixed in a 1:1 ratio to generate the mixed culture biocatalyst with a final relative OD of 20 for each component. The biotransformation was performed in 8 ml screw cap glass vials, with 1 ml of the mixed culture biocatalyst and 100 U (µmol/min for ~3 mg of powder) of PfeI added as a lyophilized powder. The substrate was added last from a stock

solution to obtain a final concentration of 5 mM. The biotransformation was then performed at 30 or 33 °C. Samples were taken after 0, 1, 5, and 24 hours. Analysis was performed with GC or HPLC.

5.4 Chemo-enzymatic one-pot reaction using a whole-cell mixed culture biocatalyst with PAMO /TmCHMO, AlkJ, and PfeI

The mixed culture biocatalyst was prepared according to SI 5.3. An appropriate volume of sodium phosphate buffer (100 mM, pH 7.4) was added for the final resuspension to obtain a final OD_{590} of 80. The cell suspensions were then mixed in a 1:1 ratio to obtain the mixed culture biocatalyst with a relative OD of 40 for each component. For the chemo-enzymatic one-pot reaction, 0.2 ml of the reaction mixture obtained after the Wacker oxidation was diluted with 0.2 ml of H_2O. The mixture's pH was then adjusted by adding 2 N NaOH (usually 10-20 µl) to 7 – 8. Subsequently, water was added to a final volume of 0.5 ml. To this, 0.5 ml of the mixed culture biocatalyst was added together with PfeI 100 U (µmol/min for ~3 mg of powder), as a lyophilized powder. Biotransformation was then performed at 30 or 33 °C (for TmCHMO or PAMO, respectively). Samples were taken after 0, 1, 5, and 24 hours. Analysis was performed with GC or HPLC.

5.5 Optimized procedure for the chemo-enzymatic one-pot reaction using a whole-cell mixed culture biocatalyst with PAMO /TmCHMO, AlkJ, and PfeI

The mixed culture biocatalyst was prepared according to SI 5.3. An appropriate volume of sodium phosphate buffer (200 mM, pH 7.4) was added for the final resuspension to obtain a final OD_{590} of 80. The cell suspensions were then mixed in a 1:1 ratio to obtain the mixed culture biocatalyst with a relative OD of 40 for each component. For the chemo-enzymatic one-pot reaction, 0.2 ml of the reaction mixture obtained after the Wacker oxidation was diluted with 0.25 ml of PB buffer (200 mM, pH 7.4), and 25 µl EtOH and H_2O were then added. To this mixture, 0.5 ml of the mixed culture biocatalyst was added together with 100 U PfeI (µmol/min for ~3 mg of powder) as a lyophilized powder. Biotransformation was then performed at 30 or 33 °C (for TmCHMO or PAMO, respectively). Samples were taken after 0, 1, 5, and 24 hours. Analysis was performed with GC or HPLC.

5.6 Preparation of lyophilized cells containing PfeI

A pre-culture of an *E. coli* strain containing a pGaston::PfeI vector was cultivated in a 15 ml flacon tube charged with 5 ml LB-Miller medium and supplemented with Ampicillin (5 µl, 100 mg/ml). The culture was grown for 19 hours at 190 rpm. Subsequently, a 1000 ml baffled flask charged with 200 ml TB-medium and supplemented with Ampicillin (200 µl, 100 mg/ml) was inoculated with 1 % (v/v) of the pre-culture. The culture was grown at 37 °C at 170 rpm until an OD_{590} of 0.29 was reached. Protein production was induced by the addition of 20 % L-Rhamnose (0.2 % v/v). The cells were then incubated for 3 hours at 37 °C, followed by harvesting through centrifugation (6000 rcf. 10 min.). The cell pellet

was resuspended in sterile water (10 ml) in a 15 ml Falcon tube. After snap-freezing the suspension in liquid nitrogen, the cells were then lyophilized for 24 hours. The resulting off-white powder was stored at –80 °C. The esterase activity was determined photometrically *via* a p-nitrophenol acetate assay [1].

5.7 ADO chemo-enzymatic one-pot biotransformation and substrate scope

The whole-cell biocatalyst was prepared according to the general procedure for protein production and biotransformation. For the chemo-enzymatic one-pot reaction, the product (**1b-8b**) obtained after the isomerization was dissolved in EtOH to get a solution of 0.5 M and added to 1 ml of the whole cell suspension (OD_{590} 30) to obtain a final concertation of 5 - 8 mM. The biotransformation was then performed at 50 °C, 220 rpm. Samples were taken after 0, 1, 5, and 24 hours and analyzed *via* GC (Figure 3). ADO only showed activity toward substrate **7b** (and **8b** after hydrolysis of the acetate) The desired aldehyde vanillin (**7f**) was formed with a yield of **50 %** (GC) after 5 hours. For **8b,** solubility issues of the substrate in the aqueous medium led to recovery issues in the 0- and 1-hour samples, as seen in the GC trace (Figure 1 b). Vanillin **7f** was obtained with a yield of **61 %** (GC) after 24 hours.

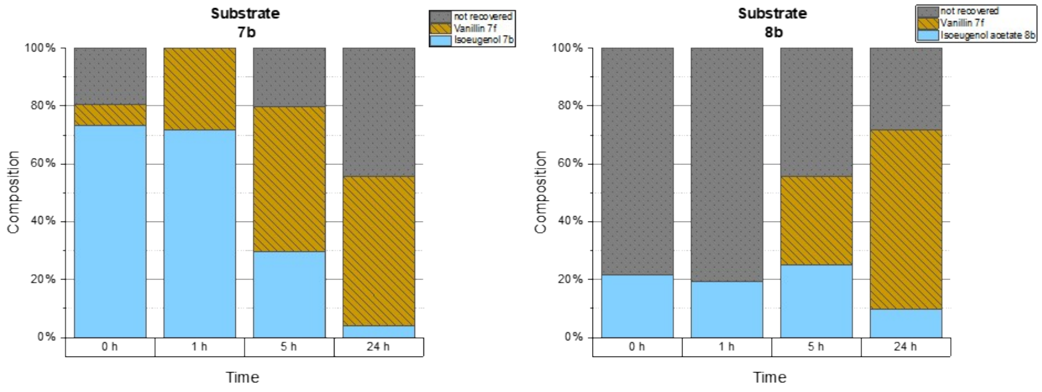

*Figure 1 Chemo-enzymatic biotransformation with **7b** and **8b** and ADO. Biotransformation with **7b** was performed with 8 mM of the substrate and 5 mM for substrate **8b**.*

5.8 ADO biotransformation with 4-(prop-2-en-1-yl)phenol

To test the hypothesis that the activity and substrate acceptance of ADO strongly depends on the stabilizing effect of hydrogen bonding with the para hydroxyl group of the substrate anethol **4b** was demethylated and subjected to ADO in a preparative scale experiment (30 ml OD_{590}, 30, 5 mM). The biotransformation was performed at 50 °C with 220 rpm. After 24 hours, the product was extracted with ethyl acetate, and the solvent was dried over Na_2SO_4 and filtered. After removing the solvent under reduced pressure *via* rotary evaporation, the residue was analyzed via ^{1}H-NMR (Figure 2). The obtained NMR spectrum (red) is shown overlayed with the spectrum of the starting material (cyan). The characteristic aldehydic proton signal at 9.85 ppm is clearly visible, and the aromatic signals at 7.80 and 6.95 ppm indicate a partial oxidation to the corresponding aldehyde.

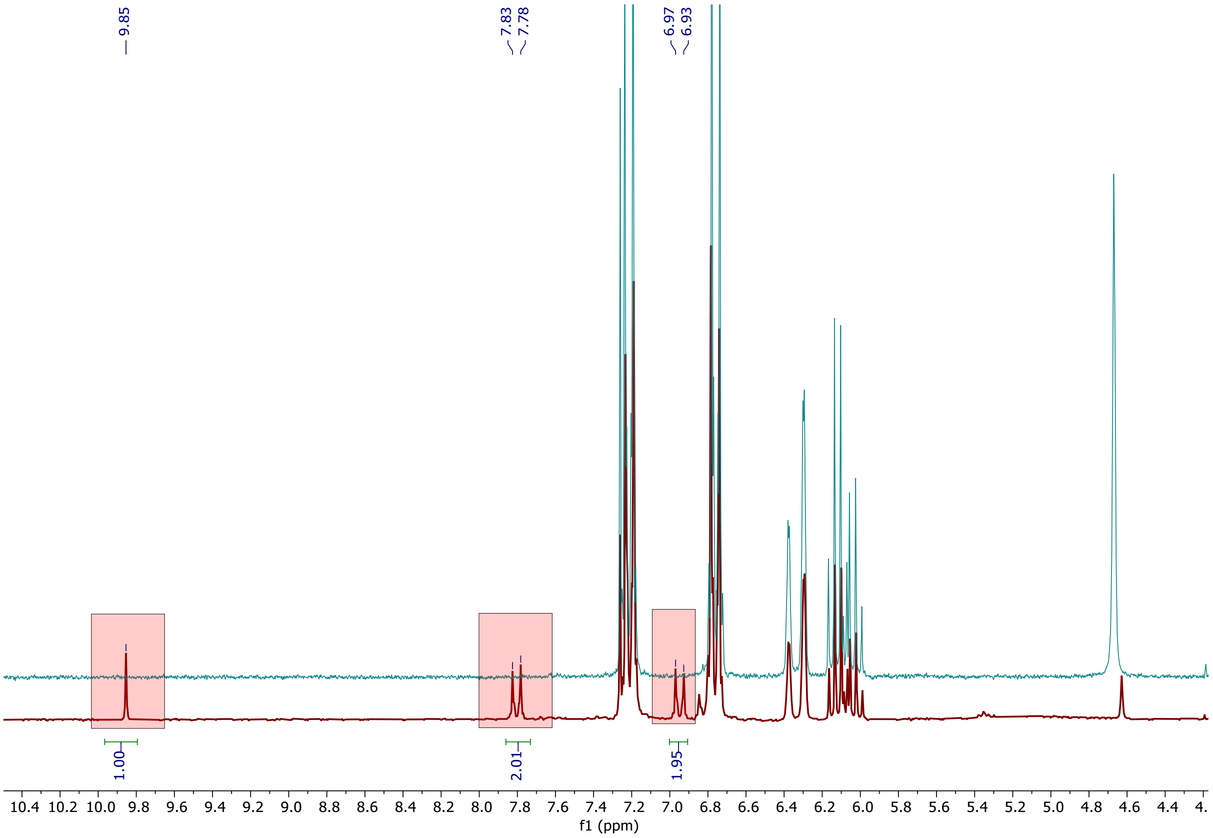

Figure 2: ^{1}H-NMR of the product of the biotransformation of 4-(prop-2-en-1yl)phenol with ADO (red) in comparison with the ^{1}H-NMR of the starting material (cyan).

5.9 AlkJ, PAMO, and TmCHMO substrate scope

To test for the substrate scope of the Baeyer-Villiger-monooxygenases PAMO and TmCHMO and the alcohol dehydrogenase AlkJ, small-scale biotransformations were performed with the purified substrate. The whole-cell biocatalyst was prepared according to the general procedure for protein production and biotransformation. For this, 1 ml of the whole-cell suspension was filled into an 8 ml screw-capped glass vial, followed by the addition of a solution of the respective substrate (10 µl, 0.5 M in EtOH) to obtain a final concentration of 5 mM. The biotransformation was then performed at 30 °C for TmCHMO and AlkJ and 37 °C for PAMO at 220 rpm. Samples were taken after 0, 1, 5, and 24 hours and analyzed *via* GC (Figure 3-5).

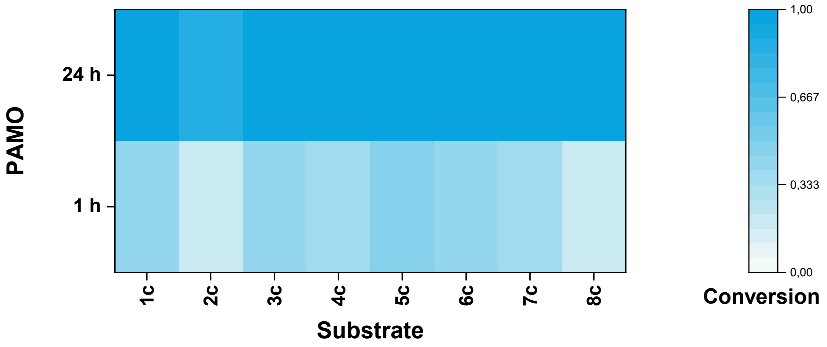

*Figure 3: Conversions of the substrates **1c** – **8c** after 1h and 24 h with PAMO. All substrates were successfully oxidized to the desired products.*

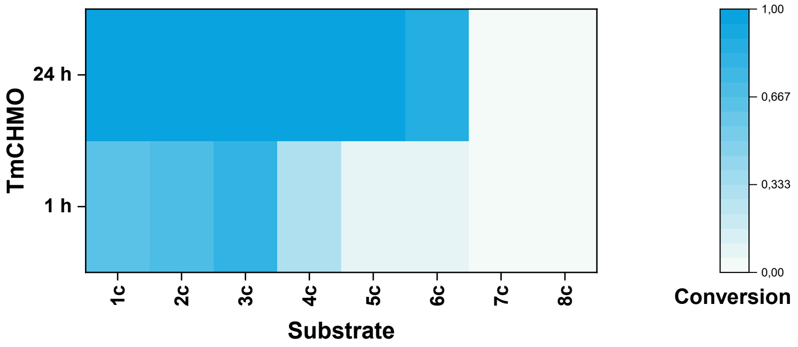

*Figure 4: Conversions of the substrates **1c** – **8c** after 1h and 24 h with TmCHMO. All substrates except **7c** and **8c** were successfully oxidized to the desired products.*

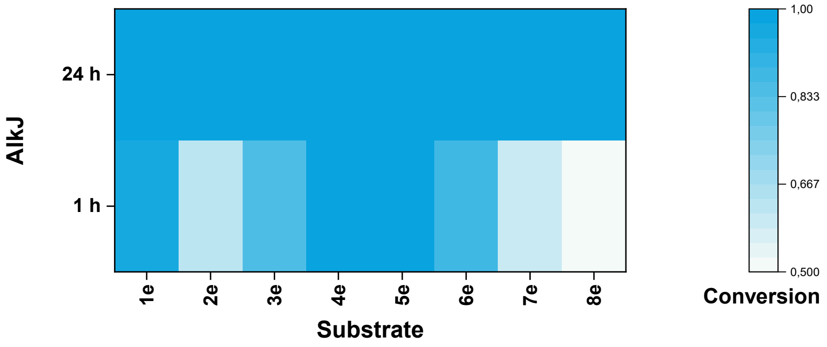

*Figure 5: Conversions of the substrates **1e** – **8e** after 1h and 24 h with AlkJ. All substrates were successfully oxidized to the desired products.*

5.10 PAMO, PfeI and AlkJ enzymatic cascade with purified substrate -GC analysis

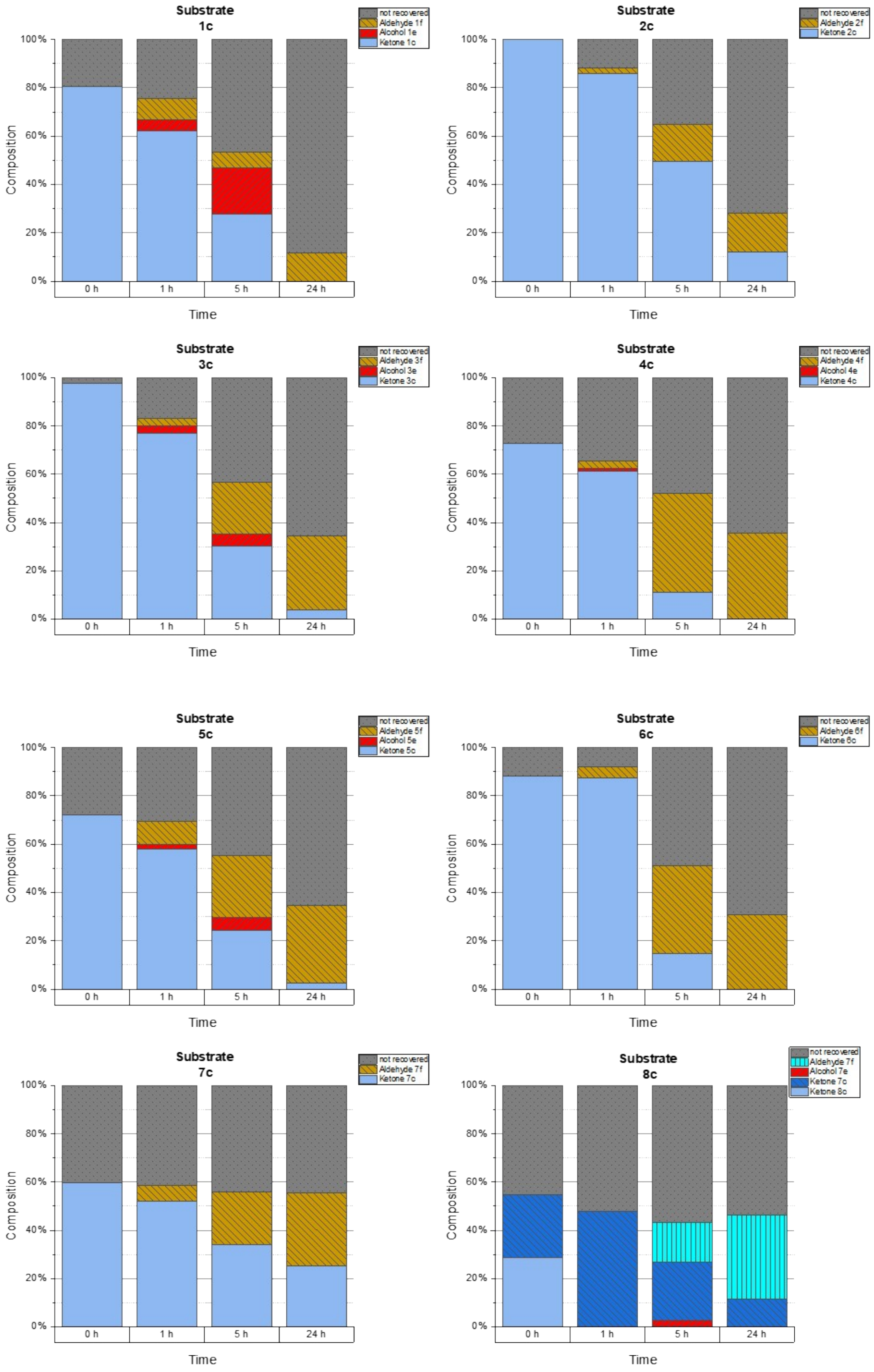

*Figure 6: Results of the biotransformation using a whole-cell mixed culture biocatalyst (PAMO, AlkJ and, PfeI) with substrates **1c-8c** (5 mM). The substrate dissolved in EtOH (0.5 M) was added to the suspension. Samples were taken after 0, 1, 5, and 24 hours and analyzed via GC. Overoxidation of the formed aldehyde was observed (not quantified with GC).*

5.11 PAMO, PfeI and AlkJ chemo-enzymatic cascade -GC analysis

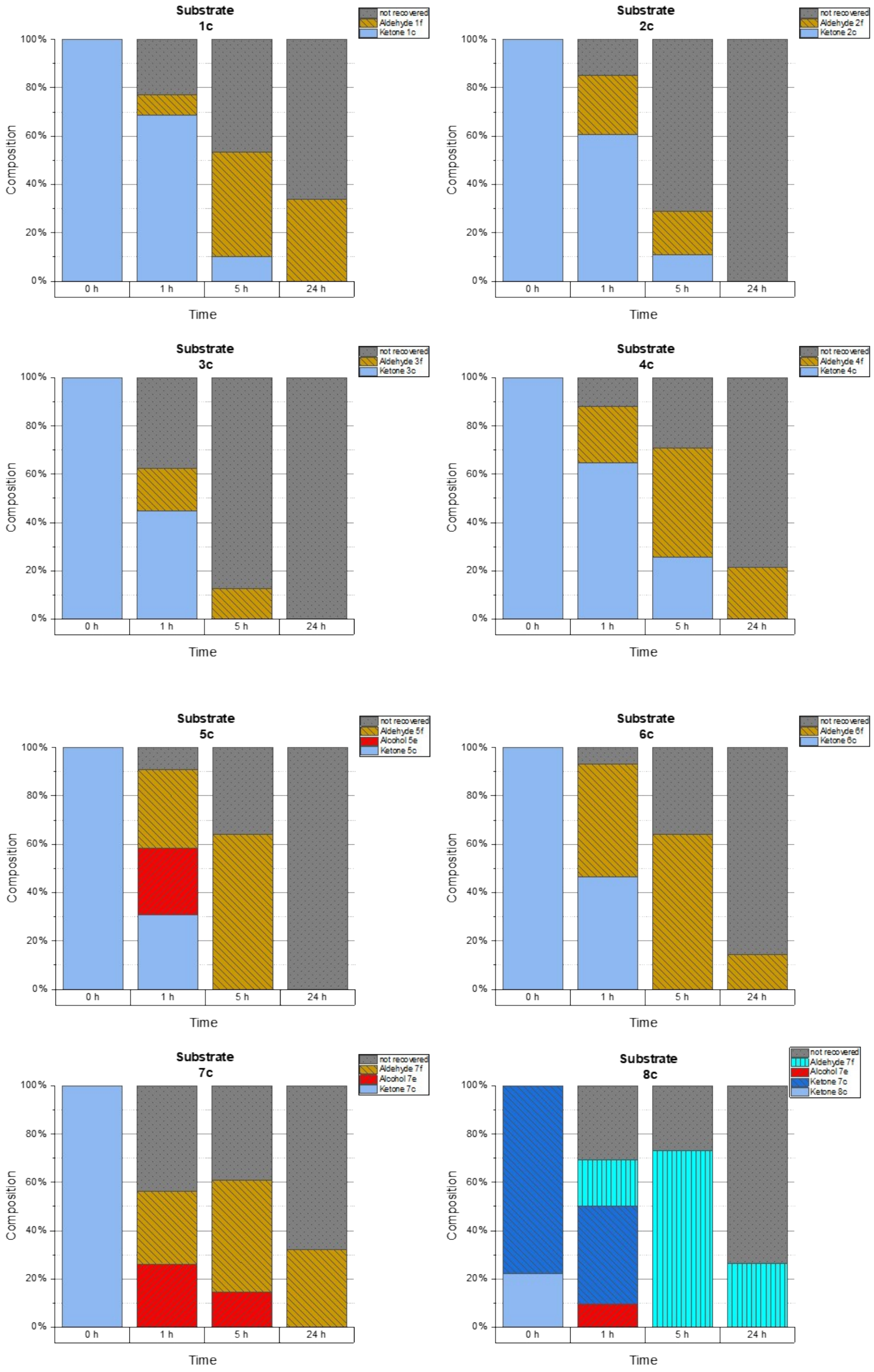

*Figure 7 Results of the chemo-enzymatic one-pot reaction using a whole-cell mixed culture biocatalyst (PAMO, AlkJ and, PfeI) with substrates **1c-8c** (max. ~1.5-4 mM). The substrate was added as a solution in the form of the reaction mixture obtained after the Wacker oxidation. Samples were taken after 0, 1, 5, and 24 hours and analyzed via GC. Overoxidation of the formed aldehyde was observed (not quantified with GC).*

5.12 TmCHMO, PfeI and AlkJ chemo-enzymatic cascade -GC analysis

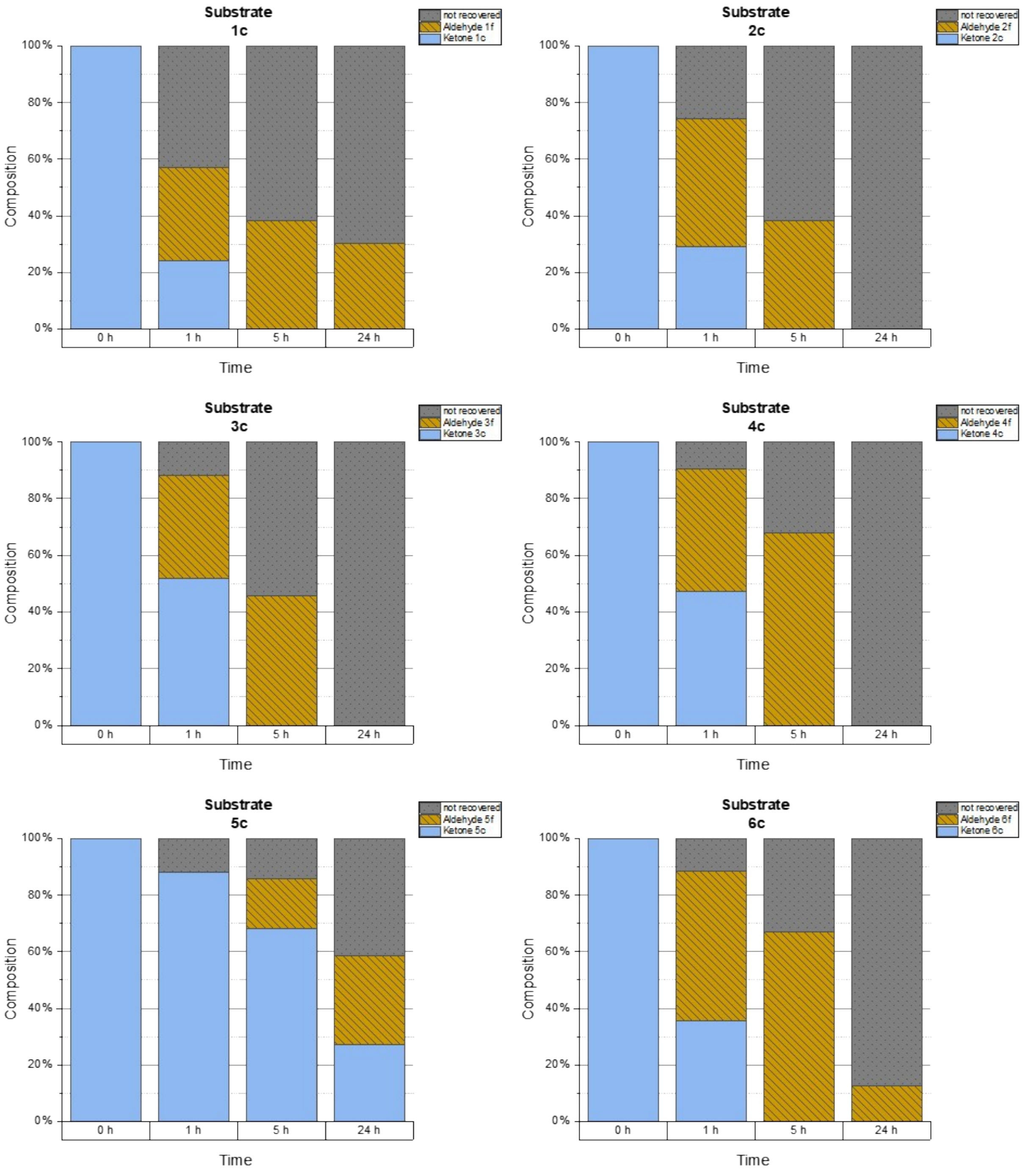

*Figure 8: Results of the chemo-enzymatic sequential one-pot reaction using a whole-cell mixed culture biocatalyst (TmCHMO, AlkJ, and PfeI) with substrates **1c-6c** (~1.5-4 mM). The substrate was added as a solution in the form of the reaction mixture obtained after the Wacker oxidation (**1a-6a**). Samples were taken after 0, 1, 5, and 24 hours and analyzed via GC. Overoxidation of the formed aldehyde was observed (not quantified with GC).*

5.13 Optimization of the chemo-enzymatic sequential reaction cascade –(U)HPLC analysis

The Insufficient mass recovery encountered in GC analysis (as seen in 5.10 – 5.12) suggested the formation of a side product that was not identifiable with the analytical method deployed. Exchanging the analytical system from GC to HPLC analysis revealed the formation of major amounts of the corresponding carboxylic acid during the enzymatic cascade, making the optimization of the enzymatic cascade necessary. We discovered that increasing the buffer concentration and adding 2.5 vol% EtOH into the biotransformation mixture could almost entirely suppress the formation of undesired carboxylic acid (Figure 9). The phenotype of the colony picked for protein expression also seemed to have a noticeable impact on the system's efficiency. As an undesired side effect, the addition of EtOH led to a slight decrease in the transformation speed (Figure 9) The optimized protocol for the biotransformation can be found in chapter 5.5.

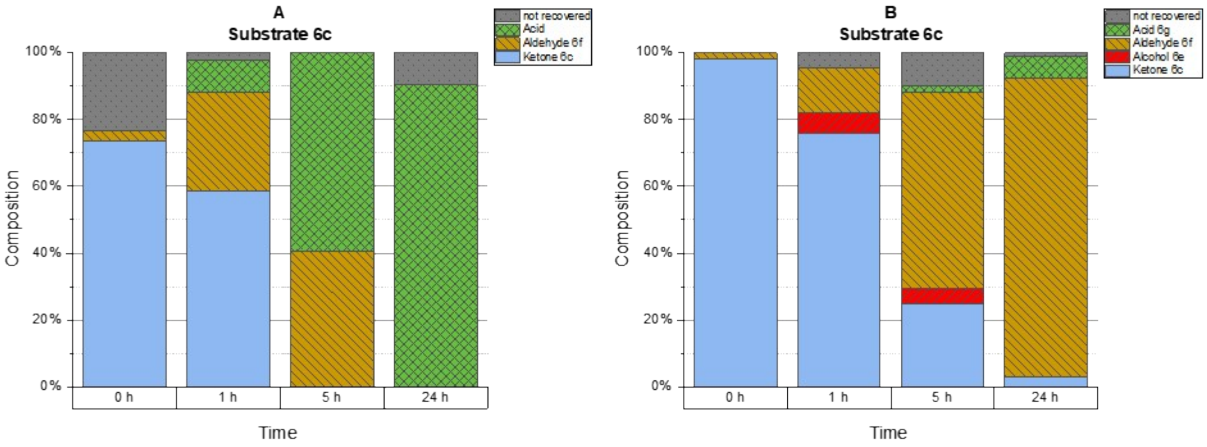

Figure 9: Difference in the product distribution obtained after the chemo-enzymatic reaction sequence with PAMO/AlkJ and PfeI. A) The standard conditions (see 5.4) led to the exclusive formation of the corresponding carboxylic acid after 24 hours. B) The optimized conditions (see 5.5) led to a sharp decrease in acid formation.

5.14 PAMO, PfeI, and AlkJ chemo-enzymatic cascade –(U)HPLC analysis

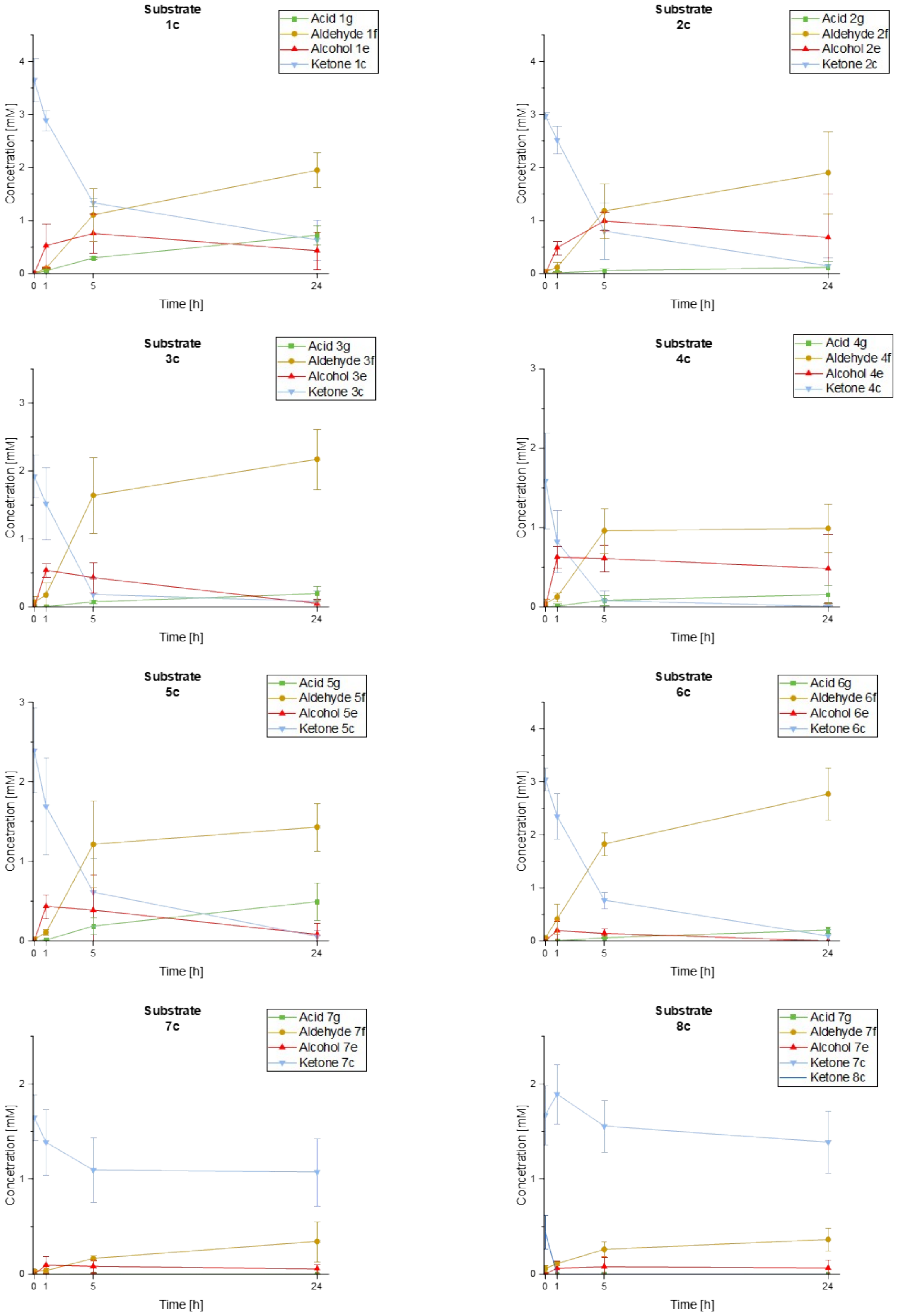

*Figure 10 Results of the optimized chemo-enzymatic sequential one-pot reaction using a whole-cell mixed culture biocatalyst (PAMO, AlkJ, and PfeI) with substrates **1c- 8c** (~1.3- 4 mM). The substrate was added as a solution in the form of the reaction mixture obtained after the Wacker oxidation (**1a- 8a**). Samples were taken after 0, 1, 5, and 24 hours and analyzed via (U)HPLC. Overoxidation of the formed aldehyde was observed in small amounts.*

5.15 Large-scale biotransformation using 4a

For the preparative-scale biotransformation, the Wacker oxidation was performed with allyl anisole **4a** (37 mg) under standard conditions with 9.5 ml of H_2O and 0.5 ml ACN as co-solvent. After the complete consumption of the starting material was confirmed, the pH of the solution was adjusted to 7.4 through the addition of 2 N NaOH. Removal of the precipitates via centrifugation led to a yellow solution. This solution was added to 50 ml of the mixed culture biocatalyst (OD 20 TmCHMO, OD 20 AlkJ) together with 100 U (µmol/min for ~3 mg of powder) of PfeI in a 250 ml flask equipped with a rubber septum to prevent the evaporation of the reaction components. The biotransformation was conducted at 30 °C and 220 rpm. Samples were taken after 0, 1, 5, and 19 hours. At the 0 h mark of the biotransformation the yield of the Wacker-oxidation was determined to be 71 %. After GC analysis (Figure 11) confirmed complete consumption of the starting material, the suspension was centrifuged, the supernatant transferred into a separating funnel, and extracted with EtOAc (3 x 30 ml). Removal of the solvent resulted in a brown residue (31 mg) which was filtered through a pad of silica. ^{1}H-NMR analysis of the residue revealed a mixture of the desired aldehyde (34 % after four steps) and the corresponding carboxylic acid obtained after overoxidation (Figure 12).

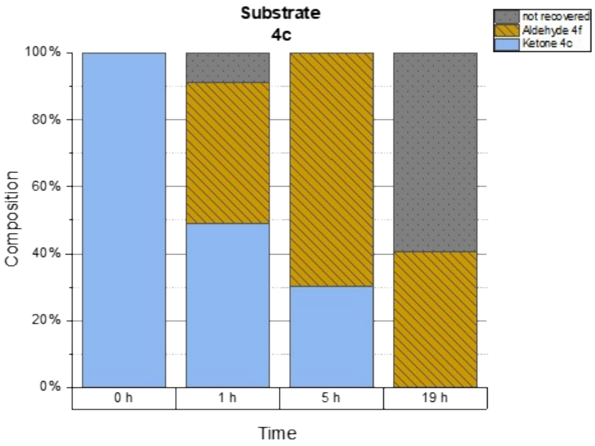

Figure 11. GC-trace of the preparative scale biotransformation

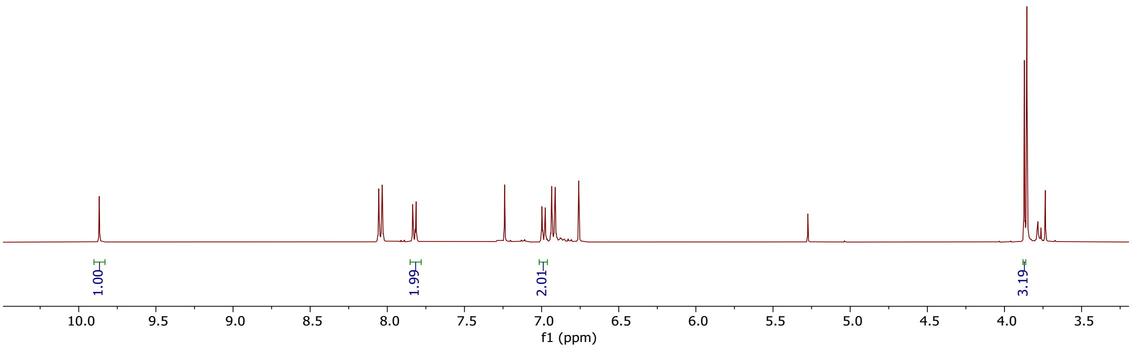

Figure 12: ^{1}H-NMR of the crude obtained from the reaction sequence.

5.16 General procedure for the Wacker-oxidation of phenylpropenes[2]

A reaction vessel equipped with a septum and magnetic stirring was charged with $Pd(TFA)_2$, NaTFA (0.2 equiv.), $FeCl_3$ ($Fe_2(SO_4)_3$. The vessel was then evacuated and flushed with Argon utilizing standard Schlenk techniques. Degassed water (three freeze-thaw cycles) was then added to the solids resulting in a yellow solution. This was followed by the slow addition of **1a**-**8a** in a solution of ACN via a syringe. The mixture was then stirred vigorously in the dark for 24 hours. After full consumption of the starting material was confirmed *via* TLC, the reaction mixture was used directly for the subsequent biotransformation or stored in the freezer for later use. For the isolation of the product, the mixture was extracted with EtOAc instead. The combined organics were washed once with saturated $NaHCO_3$ solution and brine. After drying the solution over $Na_2SO_{4,}$ and filtration, the removal of the solvent *via* rotary evaporation resulted in a brown oil. The crude product was further purified by column chromatography.

5.17 General procedure for the isomerization of phenylpropenes

A brown glass reaction vessel equipped with magnetic stirring was filled with **1a**-**8a**. To this, $PdCl_2$ was then added, and the reaction was stirred for 24 hours. The conversion was monitored via ^{1}H-NMR. The mixture was then used directly for the subsequent biotransformation or stored in the freezer for later use. For the catalyst recovery, the mixture was diluted with EtO_2 and centrifuged (5 min, 14000 rpm).

The supernatant was then removed. The residue ($PdCl_2$) was then rewashed with a fresh portion of solvent, and the procedure was repeated. The wash fractions were combined, and the solvent was removed under reduced pressure, leaving a brown oil, which was further purified via flash chromatography if necessary. The catalyst residue could be directly reused for a new batch without further treatment.

5.18 Isomerization and catalyst recovery

To demonstrate the possibility of catalyst recovery and reusability of said catalyst, the standard isomerization procedure was followed (chapter 5.17), emploing **7a** as the model substrate (1 g, 6.09 mmol) together with PdCl2 (54 mg, 5 mol%). After complete conversion was achieved, the catalyst was recovered as mentioned above, and the isomerization restarted with the recovered $PdCl_2$. This process was repeated two more times. The amount of catalyst recovered after each run can be found in table table 2.

Table 2: Amount of $PdCl_2$ recovered after each subsequent isomerization.

**run**	**catalyst (recovered [%])**	**reaction time [h]**	**Yield [%]**
(start)	54 mg	-	quant.
1	38 mg (70 %)	26	quant.
2	34 mg (89 %)	24	quant.
3	27.5 mg (80 %)	32	quant.

6. Synthesis

6.1 Synthesis of 4-(prop-2-en-1-yl)phenol[3]

A flame-dried three-necked round bottom flask equipped with a condenser and a dropping funnel was charged with $Al_{(S)}$ (0.26 g, 9.2 mmol, 2.8 equiv.) and $I_{2(S)}$. (1.41 g, 11.0 mmol, 3.3 equiv.) The atmosphere of the reaction vessel was then replaced by Argon, utilizing standard Schlenk techniques. ACN (25 ml) and DMSO (0.65 g, 8.2 mmol, 2.5 equiv.) were then added, followed by heating to 80 °C for 30 minutes under vigorous stirring. To this mixture, a solution of anethol (0.50 g, 3.3 mmol, 1.0 equiv. in 2 ml ACN) was added dropwise *via* the dropping funnel over a period of 30 minutes. The reaction was then stirred for 18 h at 80 °C. After full consumption of the starting material was confirmed via TLC, the heating was removed, allowing the mixture to cool down to room temperature. The reaction was then quenched via the addition of 2 N HCl, and the product was extracted with EtOAc (4 x 10 ml). The combined organic phases were then washed with aq. $Na_2S_2SO_3$ and brine. The solution was then dried over Na_2SO_4, filtered, and the solvent removed under reduced pressure. The resulting crude was then purified via flash chromatography resulting in a colorless oil, which solidified in the freezer.

Yield: 90 % (0.40 g)

^{1}H-NMR (200 MHz, DMSO) δ(ppm): 9.24 (s, 1H), 7.07 – 6.96 (m, 2H), 6.79 – 6.70 (m, 2H), 5.97 (dt, J = 16.8, 6.7 Hz, 1H), 5.15 – 4.98 (m, 2H), 3.29 (d, J = 6.8 Hz, 2H).

6.2 Isomerization

6.2.1 Synthesis of (*E*/*Z*)-1-(prop-1-en-1-yl)benzene 1b

1b was prepared according to the general procedure for the isomerization of phenylpropenes (chapter 5.17). The reaction was conducted at room temperature with $PdCl_2$ (7.5 mg, 5 mol%) and **1a** (100 mg, 0.8 mmol, 1.0 equiv.).

Yield: Quantitative (*E*/*Z*= 49:1, determined from the ^{1}H-NMR)

^{1}H-NMR (400 MHz, $CDCl_3$): *E*-isomer, δ (ppm): 7.36 – 7.22 (m, 5H), 6.39 (dq, *J* = 15.8, 1.7 Hz, 1H), 6.22 (dq, *J* = 15.8, 6.5 Hz, 1H), 1.87 (dd, *J* = 6.6, 1.6 Hz, 3H).

5.2.2. Synthesis of *(E/Z)*-1-methyl-3-(prop-1-en-1-yl)benzene 2b

2b was prepared according to the general procedure for the isomerization of phenylpropenes (chapter 5.17). The reaction was performed at room temperature with $PdCl_2$ (6.7 mg, 5 mol%) and **2a** (100 mg, 0.75 mmol, 1.0 equiv.).

Yield: Quantitative (*E*/*Z*= 49:1, determined from ^{1}H-NMR)

^{1}H-NMR (400 MHz, $CDCl_3$): *E*-isomer, δ (ppm): 7.22 – 7.11 (m, 2H), 7.14 – 7.07 (m, 1H), 7.03 – 6.96 (m, 1H), 6.36 (dq, *J* = 15.8, 1.7 Hz, 1H), 6.21 (dq, *J* = 15.7, 6.5 Hz, 1H), 2.32 (s, 3H), 1.86 (dd, *J* = 6.5, 1.6 Hz, 3H).

5.2.3. Synthesis of *(E/Z)*-1-methyl-4-(prop-1-en-1-yl)benzene 3b

3b was prepared according to the general procedure for the isomerization of phenylpropenes (chapter 5.17). The reaction was performed at room temperature with $PdCl_2$ (6.7 mg, 5 mol%) and **3a** (100 mg, 0.75 mmol, 1.0 equiv.).

Yield: 99 % conversion (*E*/*Z* 24:1, determined from ^{1}H-NMR)

^{1}H-NMR (400 MHz, $CDCl_3$): *E*-isomer, δ (ppm): 7.23 – 7.18 (m, 2H), 7.08 (d, J = 7.9 Hz, 2H), 6.35 (dq, J = 15.7, 1.7 Hz, 1H), 6.16 (dq, J = 15.7, 6.6 Hz, 1H), 2.30 (s, 3H), 1.85 (dd, J = 6.6, 1.7 Hz, 3H).

5.2.4. Synthesis of *(E/Z)*-1-methoxy-4-(prop-1-en-1-yl)benzene 4b

4b was prepared according to the general procedure for the isomerization of phenylpropenes (chapter 5.17). The reaction was performed at 40 °C with $PdCl_2$ (6.0 mg, 5 mol%) and **4a** (100 mg, 0.67 mmol, 1.0 equiv.).

Yield: Quantitative (*E*/*Z*= 24:1, determined from ^{1}H-NMR)

^{1}H-NMR (400 MHz, $CDCl_3$): *E*-isomer, δ (ppm): 7.27 – 7.21 (m, 2H), 6.85 – 6.78 (m, 2H), 6.33 (dq, *J* = 15.6, 1.7 Hz, 1H), 6.07 (dq, *J* = 15.7, 6.6 Hz, 1H), 3.78 (s, 3H), 1.84 (dd, *J* = 6.6, 1.7 Hz, 3H).

5.2.5. Synthesis of (*E*/*Z*)-1,2-dimethoxy-4-(prop-1-en-1-yl)benzene 5b

5b was prepared according to the general procedure for the isomerization of phenylpropenes (chapter 5.17). The reaction was performed at 40 °C with $PdCl_2$ (5.0 mg, 5 mol%) and **5a** (100 mg, 0.56 mmol, 1.0 equiv.).

Yield: Quantitative (*E*/*Z*= 27:2, determined from ^{1}H-NMR)

^{1}H-NMR (400 MHz, $CDCl_3$): *E*-isomer, δ (ppm): 6.89 – 6.74 (m, 3H), 6.31 (dq, J = 15.7, 1.6 Hz, 1H), 6.09 (dq, J = 15.7, 6.6 Hz, 1H), 3.87 (s, 3H), 3.85 (s, 3H), 1.85 (dd, J = 6.6, 1.7 Hz, 3H).

5.2.6. Synthesis of (*E*/*Z*)-5-(prop-1-en-1-yl)-2*H*-1,3-benzodioxole 6b

6b was prepared according to the general procedure for the isomerization of phenylpropenes (chapter 5.17). The reaction was performed at 40 °C. The reaction was performed at 40 °C with $PdCl_2$ (5.5 mg, 5 mol%) and **6a** (100 mg, 0.61 mmol, 1.0 equiv.).

Yield: Quantitative (*E*/*Z* = 63: 2, determined from ^{1}H-NMR)

^{1}H-NMR (400 MHz, $CDCl_3$): *E*-isomer, δ (ppm): 6.88 – 6.83 (m, 1H), 6.79 – 6.67 (m, 2H), 6.29 (dq, *J* = 15.7, 1.7 Hz, 1H), 6.04 (dq, *J* = 15.7, 6.6 Hz, 1H), 5.91 (s, 2H), 1.83 (dd, *J* = 6.6, 1.7 Hz, 3H).

5.2.7. Synthesis of (*E*/*Z*)-2-methoxy-4-(prop-1-en-1-yl)phenol 7b

7b was prepared according to the general procedure for the isomerization of phenylpropenes (chapter 5.17). The reaction was performed at room temperature with $PdCl_2$ (5.4 mg, 5 mol%) and **7a** (100 mg, 0.61 mmol, 1.0 equiv.).

Yield: Quantitative (*E*/*Z* = 37:1, determined from ^{1}H-NMR)

^{1}H-NMR (400 MHz, $CDCl_3$): *E*-isomer, δ (ppm): 6.88 – 6.77 (m, 3H, H2), 6.30 (dq, J = 15.7, 1.7 Hz, 1H), 6.06 (dq, J = 15.7, 6.6 Hz, 1H), 5.52 (s, 1H), 3.88 (s, 3H), 1.84 (dd, J = 6.6, 1.7 Hz, 3H).

5.2.8. Synthesis of (*E*/*Z*)-2-methoxy-4-(prop-1-en-1-yl)phenol acetate 8b

8b was prepared according to the general procedure for the isomerization of phenylpropenes (chapter 5.17). The reaction was performed at 40 °C temperature with $PdCl_2$ (4.5 mg, 5 mol%) and **8a** (100 mg, 0.48 mmol, 1.0 equiv.).

Yield: 94% conversion (*E*/*Z* = 23:1, determined from ^{1}H-NMR)

^{1}H-NMR (400 MHz, $CDCl_3$): *E*-isomer, δ (ppm): 6.95 – 6.88 (m, 3H), 6.39 (dq, J = 15.7, 1.7 Hz, 1H), 6.21 (dq, J = 15.7, 6.6 Hz, 1H), 3.86 (s, 3H), 2.33 (s, 3H), 1.91 (dd, *J* = 6.6, 1.7 Hz, 3H).

6.3 Wacker-Oxidation

6.3.1 Synthesis of phenylpropan-2-one 1c

1c was prepared according to the general procedure for Wacker oxidation (chapter 5.16). The reaction was conducted at 45 °C with $PdTFA_2$ (4.1 mg, 5mol%), **1a** (29 mg, 0.25 mmol, 1.0 equiv.) and $FeCl_3$ (60 mg, 0.375 mmol, 1.5 equiv.).

Yield: 77% (GC)

^{1}H-NMR (400 MHz, $CDCl_3$) δ(ppm): 7.36 – 7.14 (m, 5H), 3.68 (s, 2H), 2.13 (s, 3H).

5.3.2. Synthesis of 1-(3-methylphenyl)propan-2-one 2c

2c was prepared according to the general procedure for Wacker oxidation (chapter 5.16). The reaction was conducted at 30 °C with $PdTFA_2$ (4.1 mg, 5mol%), **2a** (33 mg, 0.25 mmol, 1.0 equiv.) and $Fe_2(SO_4)_3$ (150 mg, 0.375 mmol, 1.5 equiv.).

Yield: 73 % (GC)

^{1}H-NMR (400 MHz, $CDCl_3$) δ(ppm): 7.21 (t, J = 7.4 Hz, 1H), 7.07 (d, J = 7.4 Hz, 1H), 7.03 – 6.95 (m, 2H), 3.64 (s, 2H), 2.32 (s, 3H), 2.13 (s, 3H).

5.3.3. Synthesis of 1-(4-methylphenyl)propan-2-one 3c

3c was prepared according to the general procedure for Wacker oxidation (chapter 5.16). The reaction was conducted at 30 °C with $PdTFA_2$ (4.1 mg, 5mol%), **3a** (33 mg, 0.25 mmol, 1.0 equiv.) and $Fe_2(SO_4)_3$ (150 mg, 0.375 mmol, 1.5 equiv.).

Yield: 71 % (GC)

^{1}H-NMR (400 MHz, $CDCl_3$) δ(ppm): 7.17 – 7.10 (m, 2H), 7.10 – 7.04 (m, 2H), 3.63 (s, 2H), 2.32 (s, 3H), 2.12 (s, 3H).

5.3.4. Synthesis of 1-(4-methoxylphenyl)propan-2-one 4c

4c was prepared according to the general procedure for Wacker oxidation (chapter 5.16). The reaction was conducted at 45 °C with $PdTFA_2$ (4.1 mg, 5mol%), **4a** (37 mg, 0.25 mmol, 1.0 equiv.) and $FeCl_3$ (49 mg, 0.3 mmol, 1.2 equiv.).

Yield: 75% (GC)

^{1}H-NMR (400 MHz, $CDCl_3$) δ(ppm): 7.14 – 7.06 (m, 2H), 6.89 – 6.81 (m, 2H), 3.78 (s, 3H) , 3.61 (s, 2H), 2.12 (s, 3H).

5.3.5. Synthesis of 1-(3,4-dimethoxylphenyl)propan-2-one 5c

5c was prepared according to the general procedure for Wacker oxidation (chapter 5.16). The reaction was conducted at 60 °C with $PdTFA_2$ (4.1 mg, 5 mol%), **5a** (44 mg, 0.25 mmol, 1.0 equiv.) and $FeCl_3$ (60 mg, 0.375 mmol, 1.5 equiv.).

Yield: 65 % (GC)

^{1}H-NMR (400 MHz, $CDCl_3$) δ(ppm): 6.81 (d, J = 8.1 Hz, 1H), 6.77 – 6.66 (m, 2H), 3.85 (s, 6H), 3.61 (s, 2H), 2.13 (s, 3H).

5.3.6. Synthesis of 1-(2H-1,3-benzodioxol-5-yl)propan-2-one 6c

6c was prepared according to the general procedure for Wacker oxidation (chapter 5.16). The reaction was conducted at 60 °C with $PdTFA_2$ (4.1 mg, 5 mol%), **6a** (40 mg, 0.25 mmol, 1.0 equiv.) and $FeCl_3$ (49 mg, 0.3 mmol, 1.2 equiv.).

Yield: 73% (GC)

^{1}H-NMR (400 MHz, $CDCl_3$) δ(ppm): 6.78 – 6.71 (m, 1H), 6.69 – 6.59 (m, 2H), 5.93 (s, 2H), 3.58 (s, 2H), 2.13 (s, 2H).

5.3.7. Synthesis of 1-(4-hydroxy-3-methoxyphenyl)propan-2-one 7c

7c was prepared according to the general procedure for Wacker oxidation (chapter 5.16). The reaction was conducted at 60 °C with $PdTFA_2$ (8.2 mg, 10 mol%), **7a** (41 mg, 0.25 mmol, 1.0 equiv.) and $FeCl_3$ (49 mg, 0.3 mmol, 1.2 equiv.). The pH of the aqueous solution was adjusted to 1 with conc. HCl before the reaction.

Yield: 54 % (GC)

^{1}H-NMR (400 MHz, $CDCl_3$) δ(ppm): 6.86 (d, *J* = 7.8 Hz, 1H), 6.72 – 6.64 (m, 2H), 5.54 (s, 1H), 3.86 (s, 3H), 3.59 (s, 2H), 2.13 (s, 3H).

5.3.8. Synthesis of 1-(4-acetoxy-3-methoxyphenyl)propan-2-one 8b

8c was prepared according to the general procedure for Wacker oxidation (chapter 5.16). The reaction was conducted at 30 °C with $PdTFA_2$ (8.2 mg, 10 mol%), **8a** (51 mg, 0.25 mmol, 1.0 equiv.) and $FeCl_3$ (60 mg, 0.375 mmol, 1.5 equiv.).

Yield: 57 % (GC)

^{1}H-NMR (400 MHz, $CDCl_3$) δ(ppm): 6.97 (d, *J* = 7.7 Hz, 1H), 6.81 – 6.73 (m, 2H), 3.80 (s, 3H), 3.65 (s, 2H), 2.29 (s, 3H, Hb'), 2.15 (s, 3H).

7. Appendix – NMR spectra

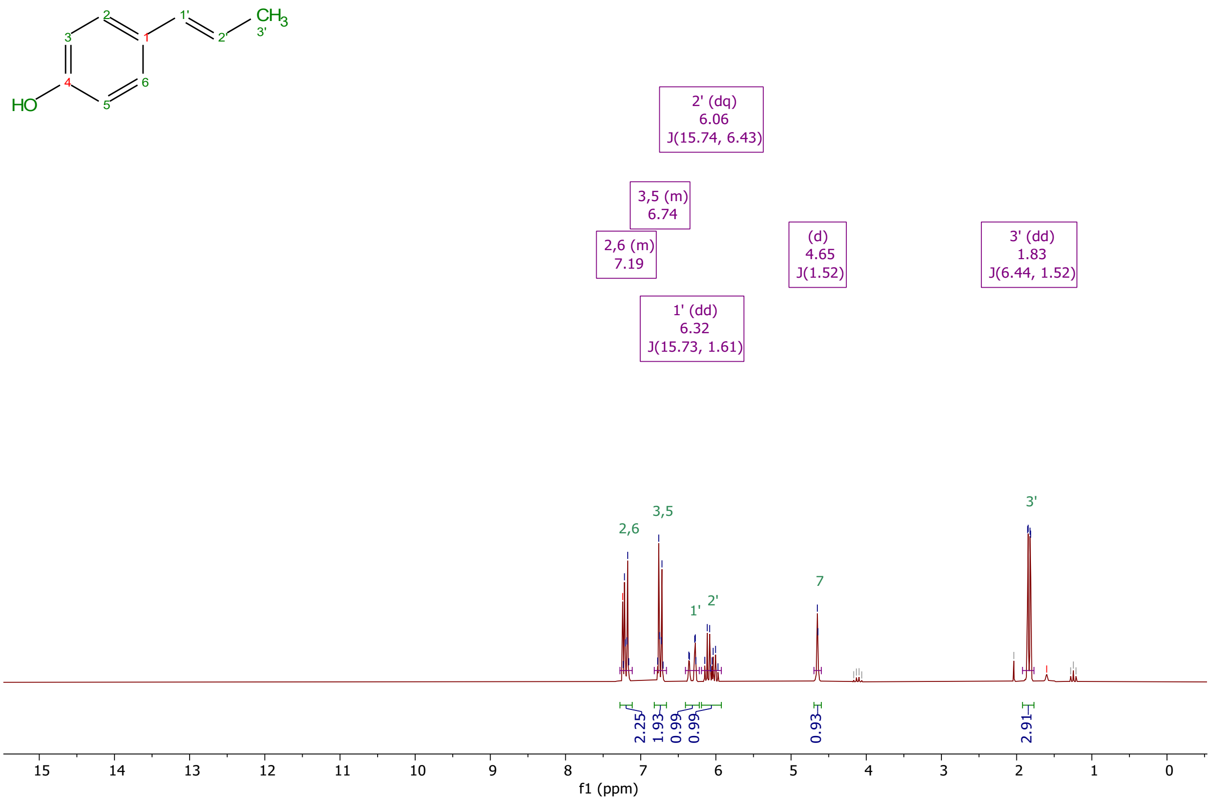

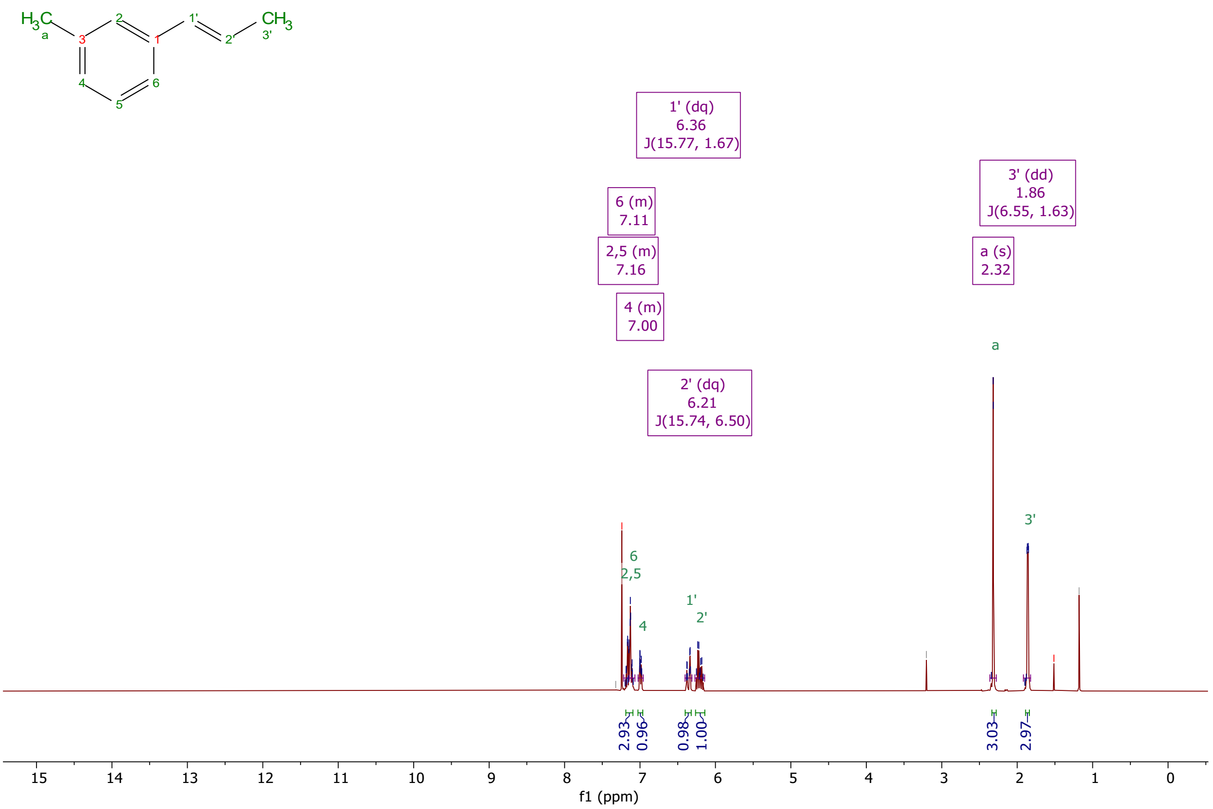
H3C
CH3
1' (dq)
6.36
J(15.77, 1.67)
6 (m)
7.11
2,5 (m)
7.16
4 (m)
7.00
2' (dq)
6.21
J(15.74, 6.50)
3' (dd)
1.86
J(6.55, 1.63)
a (s)
2.32
2.93
0.96
0.98
1.00
3.03
2.97
f1 (ppm)

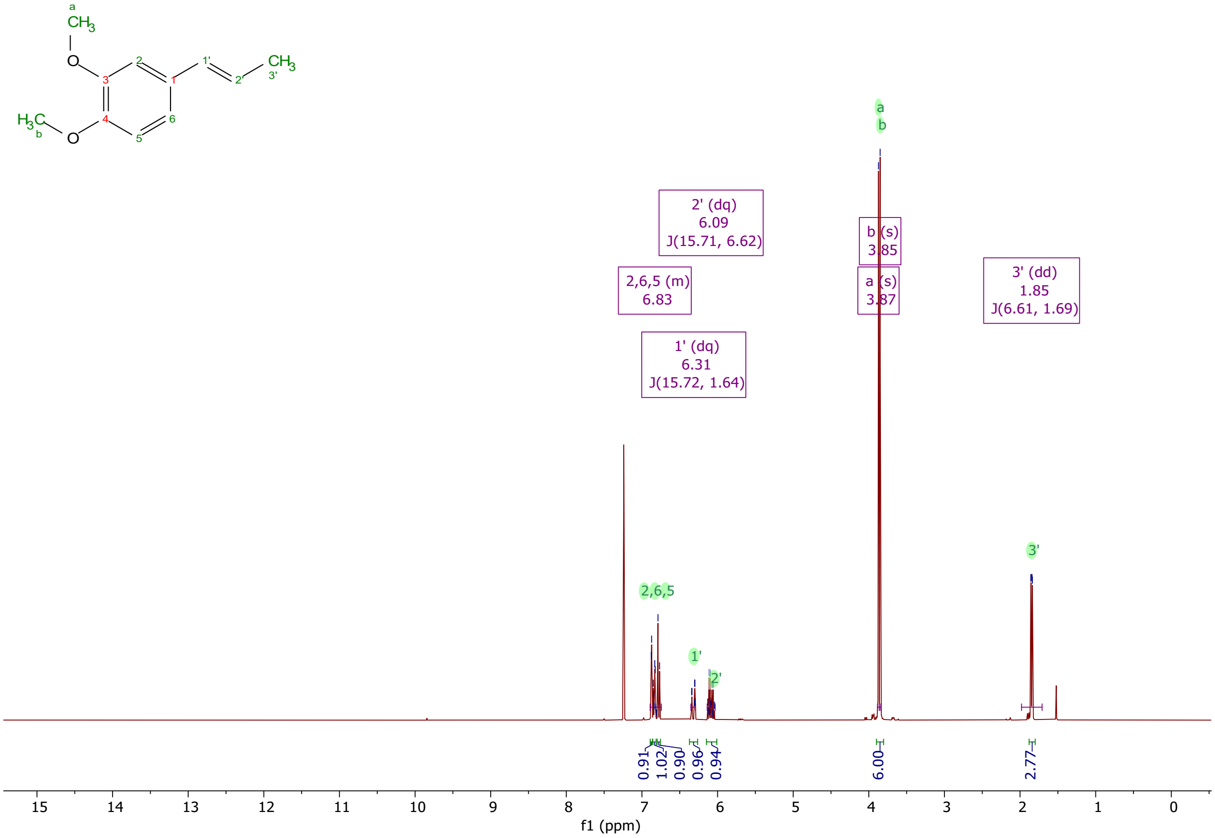
CH3
CH3
H3C
2' (dq)
6.09
J(15.71, 6.62)
2,6,5 (m)
6.83
1' (dq)
6.31
J(15.72, 1.64)
b (s)
3.85
a (s)
3.87
3' (dd)
1.85
J(6.61, 1.69)
0.91
1.02
0.90
0.96
0.94
6.00
2.77
f1 (ppm)

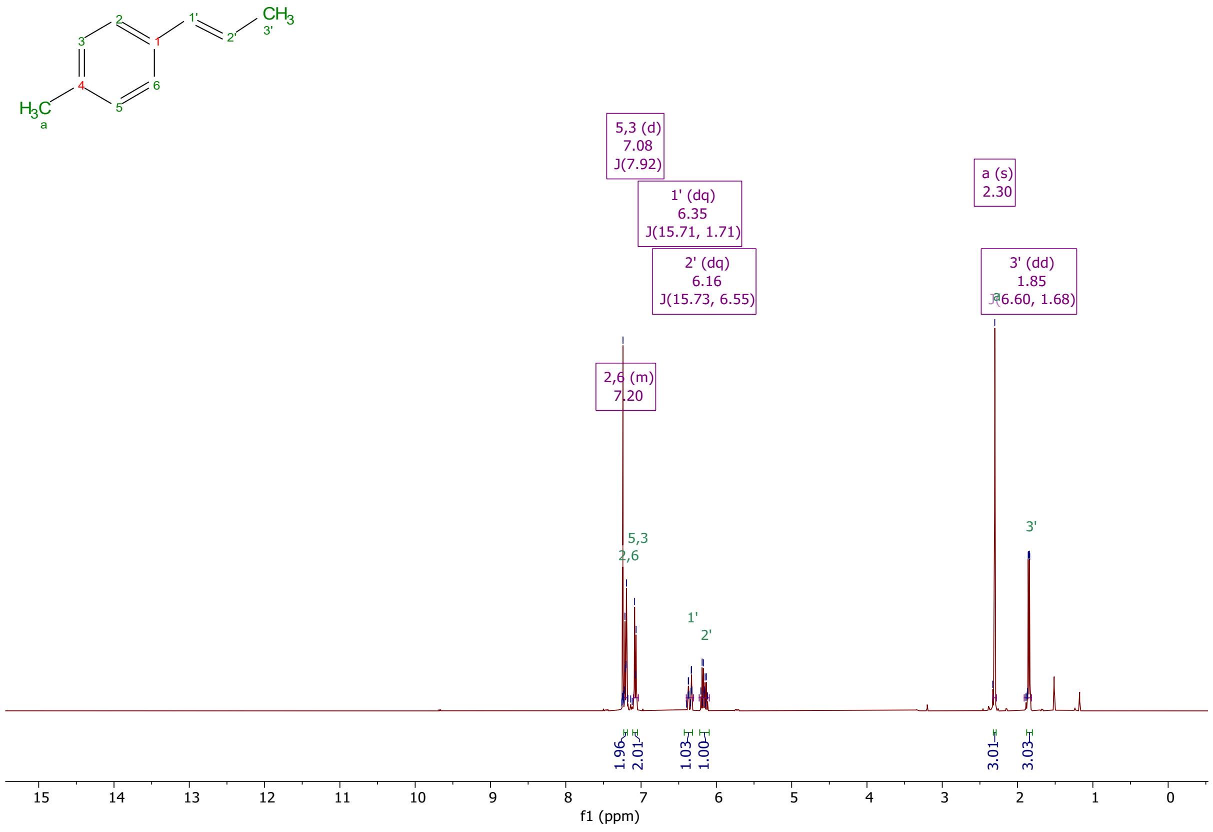
5,3 (d)
7.08
J(7.92)
1' (dq)
6.35
J(15.71, 1.71)
2' (dq)
6.16
J(15.73, 6.55)
a (s)
2.30
3' (dd)
1.85
J(6.60, 1.68)
2,6 (m)
7.20
1.96
2.01
1.03
1.00
3.01
3.03
f1 (ppm)

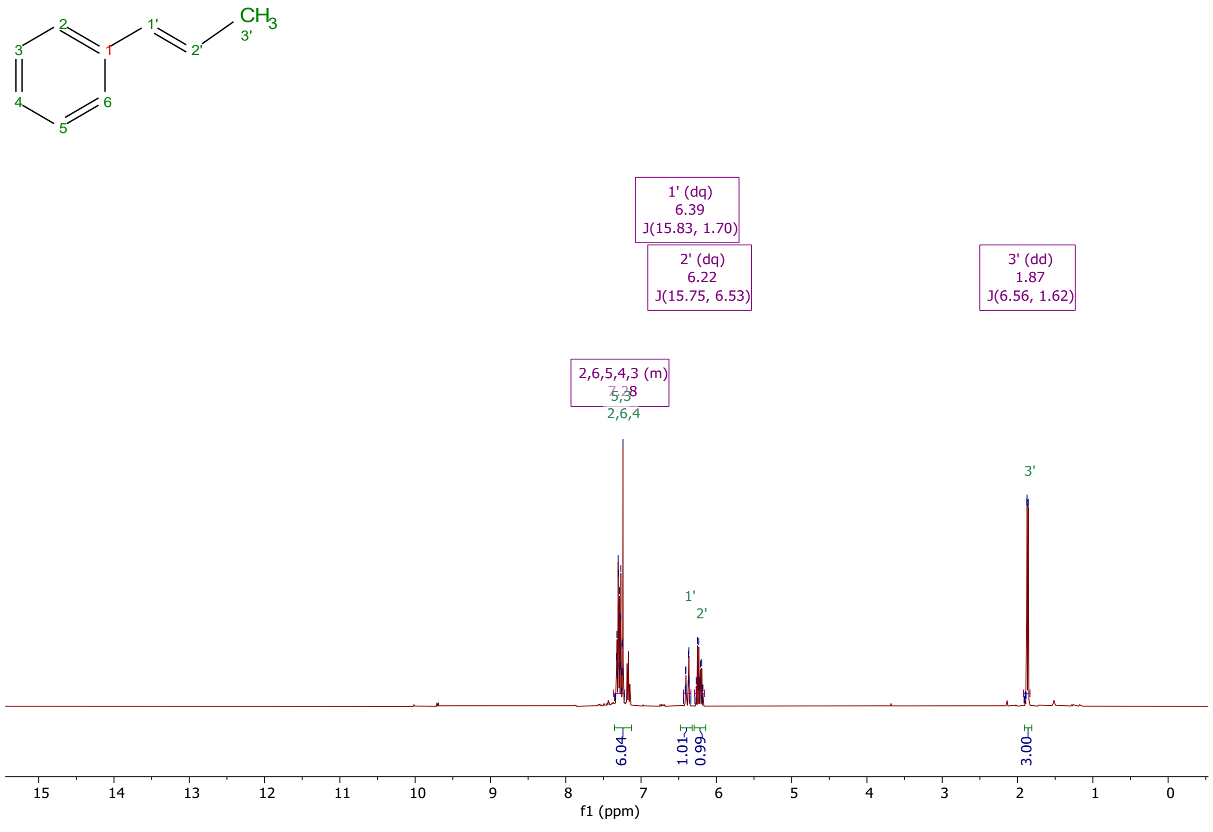
1' (dq)
6.39
J(15.83, 1.70)
2' (dq)
6.22
J(15.75, 6.53)
3' (dd)
1.87
J(6.56, 1.62)
2,6,5,4,3 (m)
6.04
1.01
0.99
3.00
f1 (ppm)

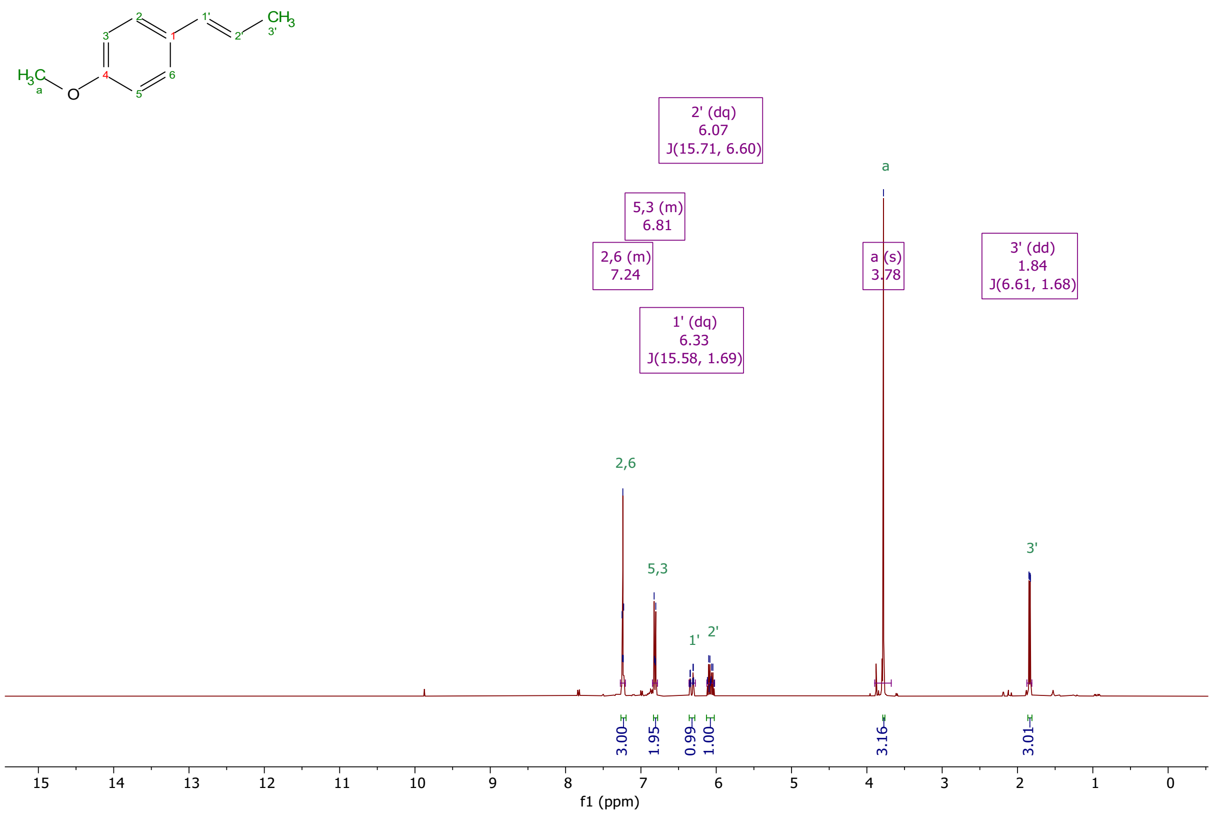
H3C
a
O
4
3
2
1
1'
2'
CH3
3'
6
5
2' (dq)
6.07
J(15.71, 6.60)
5,3 (m)
6.81
2,6 (m)
7.24
1' (dq)
6.33
J(15.58, 1.69)
a (s)
3.78
3' (dd)
1.84
J(6.61, 1.68)
a
2,6
5,3
1'
2'
3'
3.00
1.95
0.99
1.00
3.16
3.01
15
14
13
12
11
10
9
8
7
6
5
4
3
2
1
0
f1 (ppm)

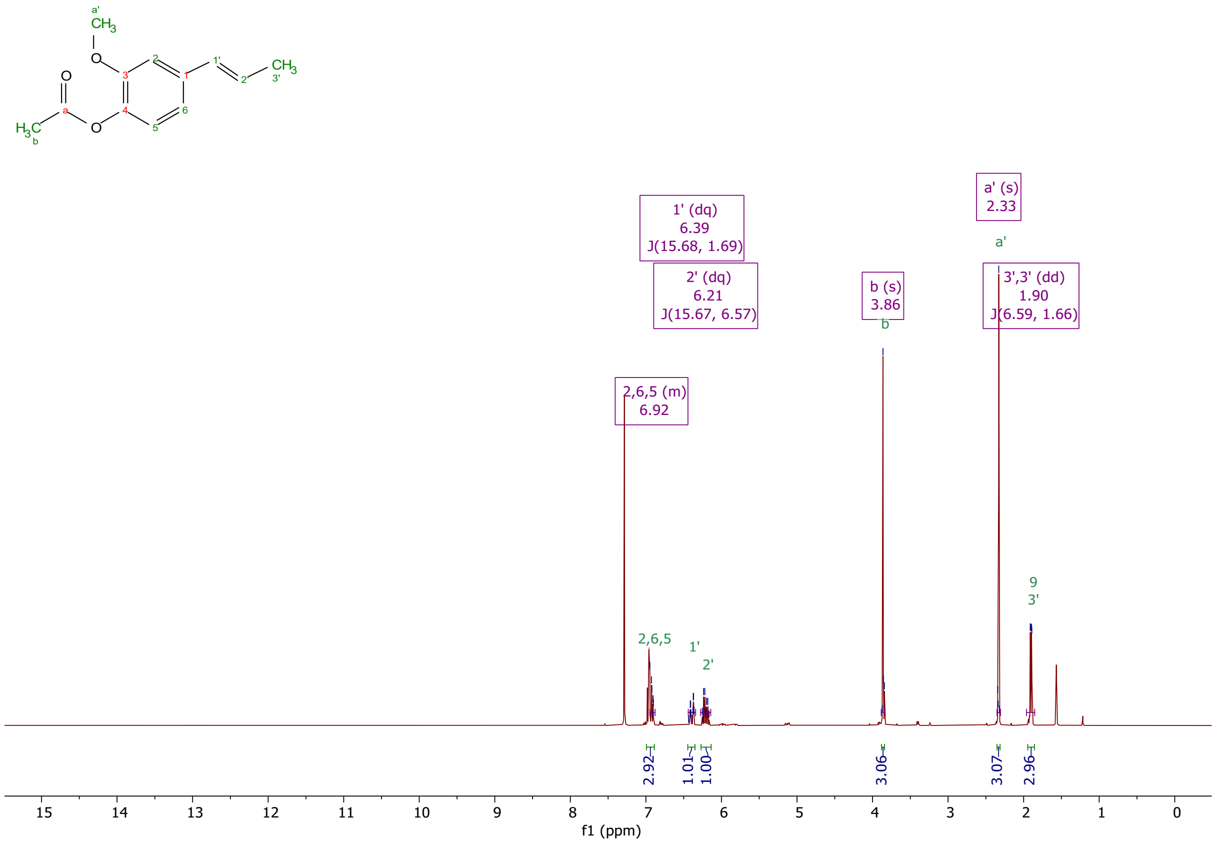
a'
CH3
O
O
a
H3C
b
O
3
4
2
1
1'
2'
CH3
3'
6
5
1' (dq)
6.39
J(15.68, 1.69)
2' (dq)
6.21
J(15.67, 6.57)
b (s)
3.86
a' (s)
2.33
3',3' (dd)
1.90
J(6.59, 1.66)
2,6,5 (m)
6.92
a'
b
9
3'
2,6,5
1'
2'
2.92
1.01
1.00
3.06
3.07
2.96
15
14
13
12
11
10
9
8
7
6
5
4
3
2
1
0
f1 (ppm)

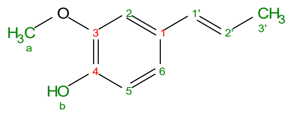
H3C
O
HO
CH3

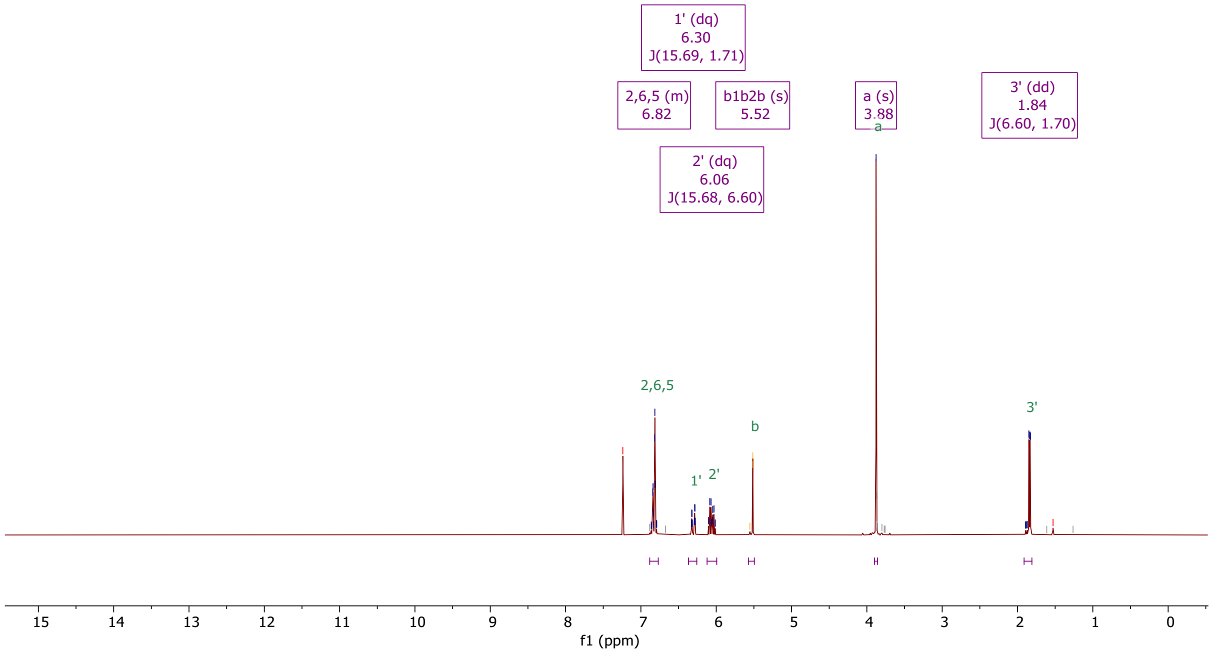
1' (dq)
6.30
J(15.69, 1.71)
2,6,5 (m)
6.82
b1b2b (s)
5.52
a (s)
3.88
3' (dd)
1.84
J(6.60, 1.70)
2' (dq)
6.06
J(15.68, 6.60)
f1 (ppm)

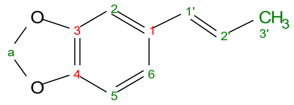
O
O
CH3

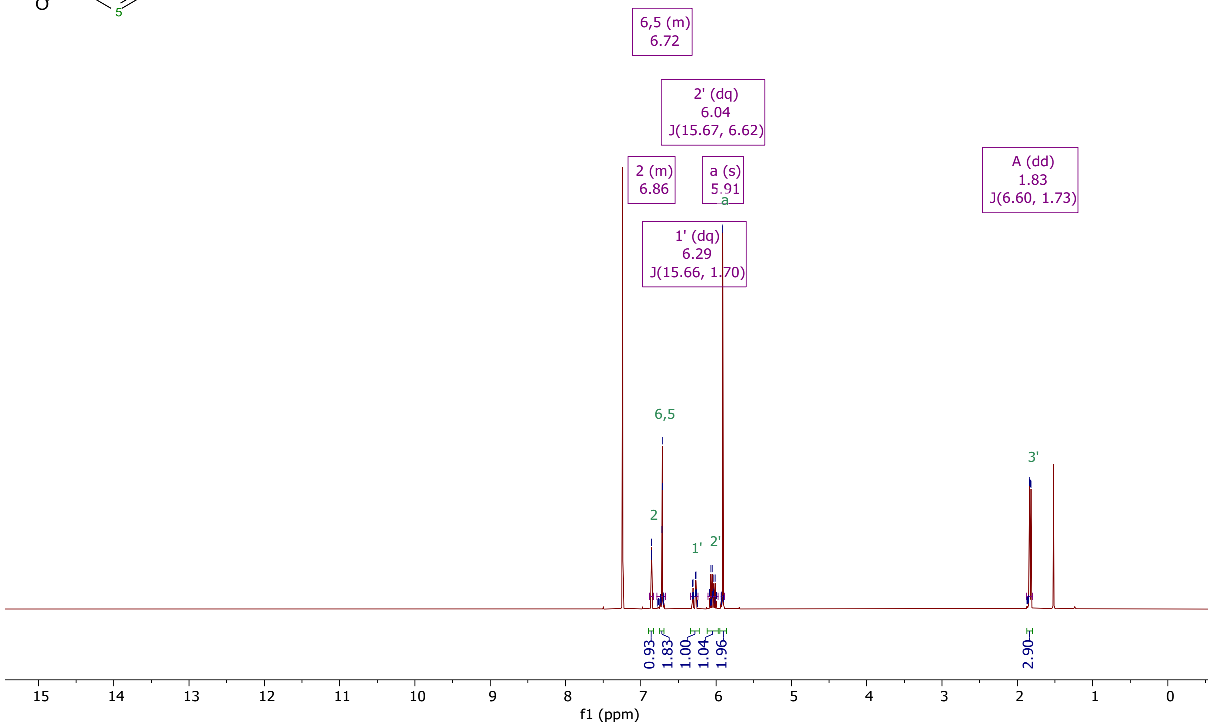
6,5 (m)
6.72
2' (dq)
6.04
J(15.67, 6.62)
2 (m)
6.86
a (s)
5.91
A (dd)
1.83
J(6.60, 1.73)
1' (dq)
6.29
J(15.66, 1.70)
f1 (ppm)

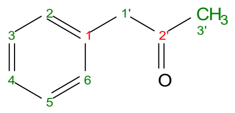
2
1'
CH_3
3'
3
1
2'
4
6
O
5

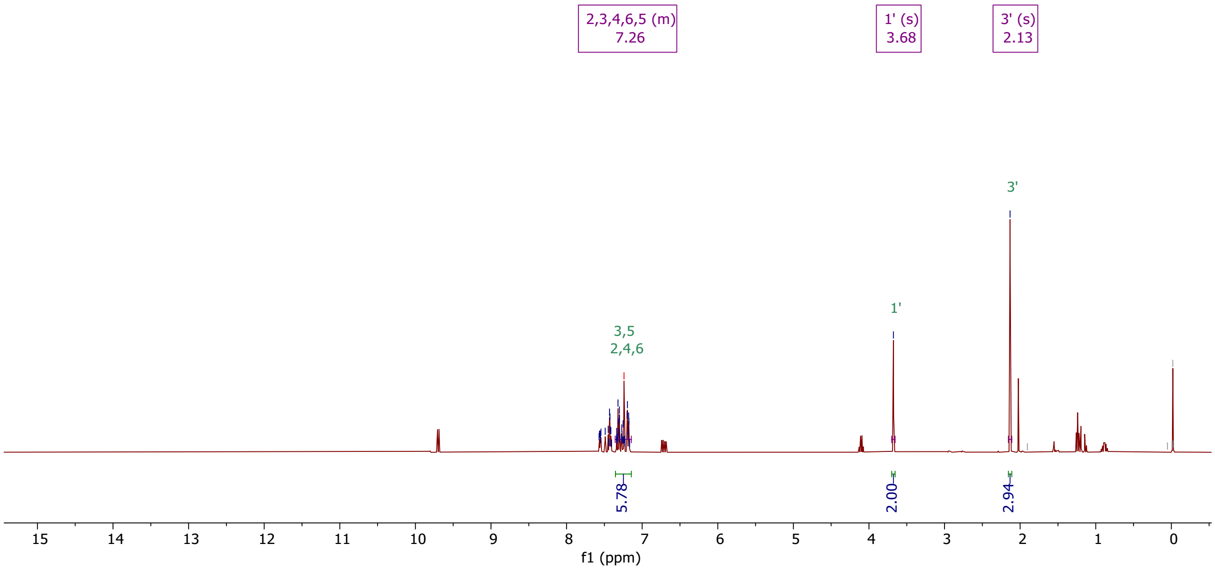
2,3,4,6,5 (m)
7.26
1' (s)
3.68
3' (s)
2.13
3'
1'
3,5
2,4,6
5.78
2.00
2.94
15 14 13 12 11 10 9 8 7 6 5 4 3 2 1 0
f1 (ppm)

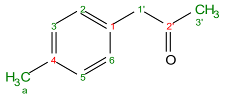
2
1'
CH_3
3'
3
1
2'
4
6
O
H_3C
a
5

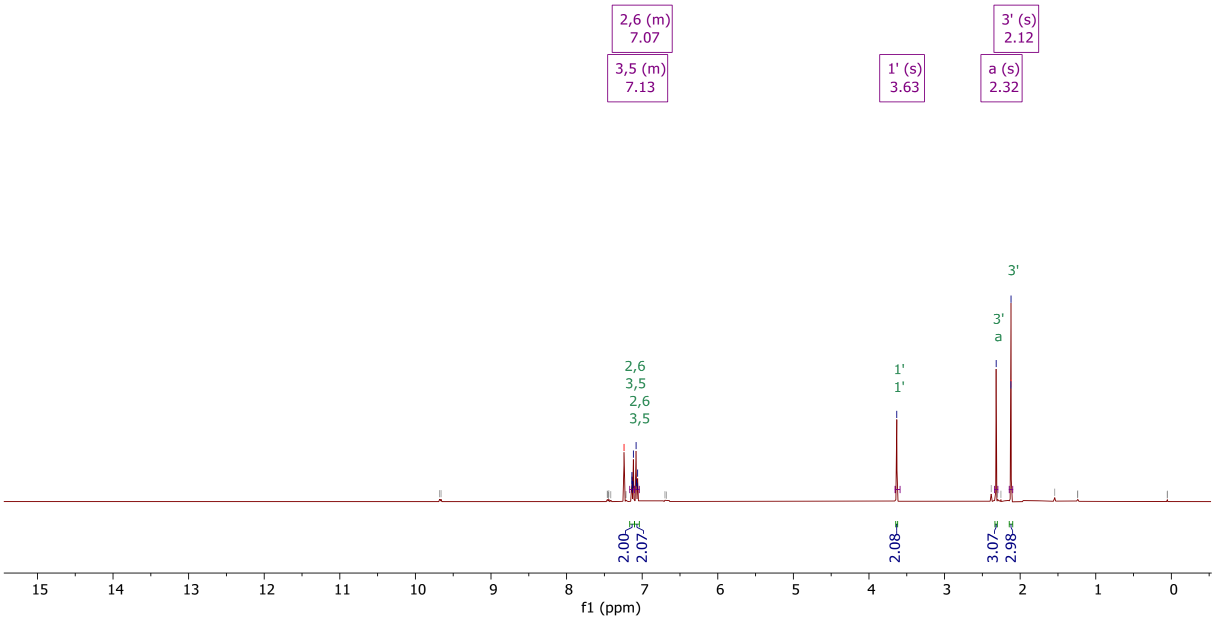
2,6 (m)
7.07
3,5 (m)
7.13
1' (s)
3.63
3' (s)
2.12
a (s)
2.32
3'
3'
a
2,6
3,5
2,6
3,5
1'
1'
2.00
2.07
2.08
3.07
2.98
15 14 13 12 11 10 9 8 7 6 5 4 3 2 1 0
f1 (ppm)

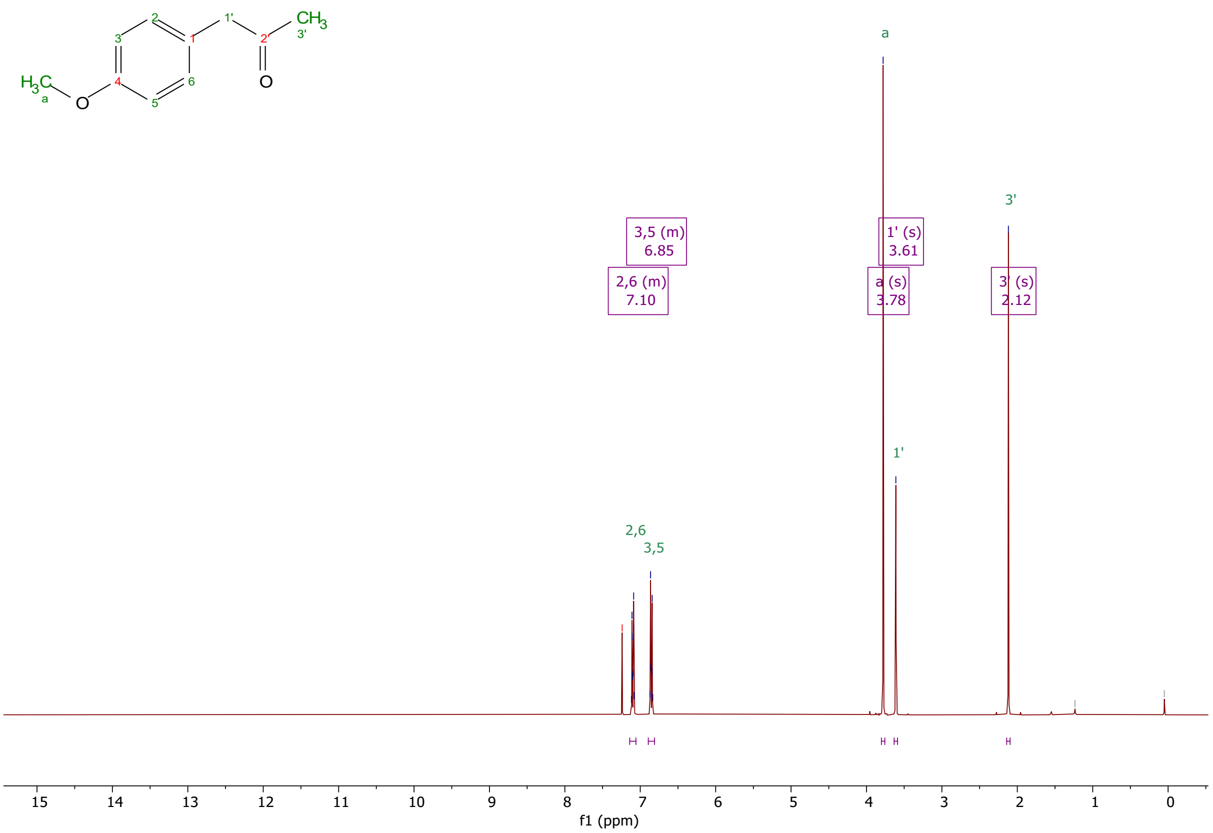
H3C a O 4 3 2 1 6 5 1' 2' CH3 3' O
a
3'
3,5 (m) 6.85
2,6 (m) 7.10
1' (s) 3.61
a (s) 3.78
3' (s) 2.12
1'
2,6
3,5
15 14 13 12 11 10 9 8 7 6 5 4 3 2 1 0
f1 (ppm)

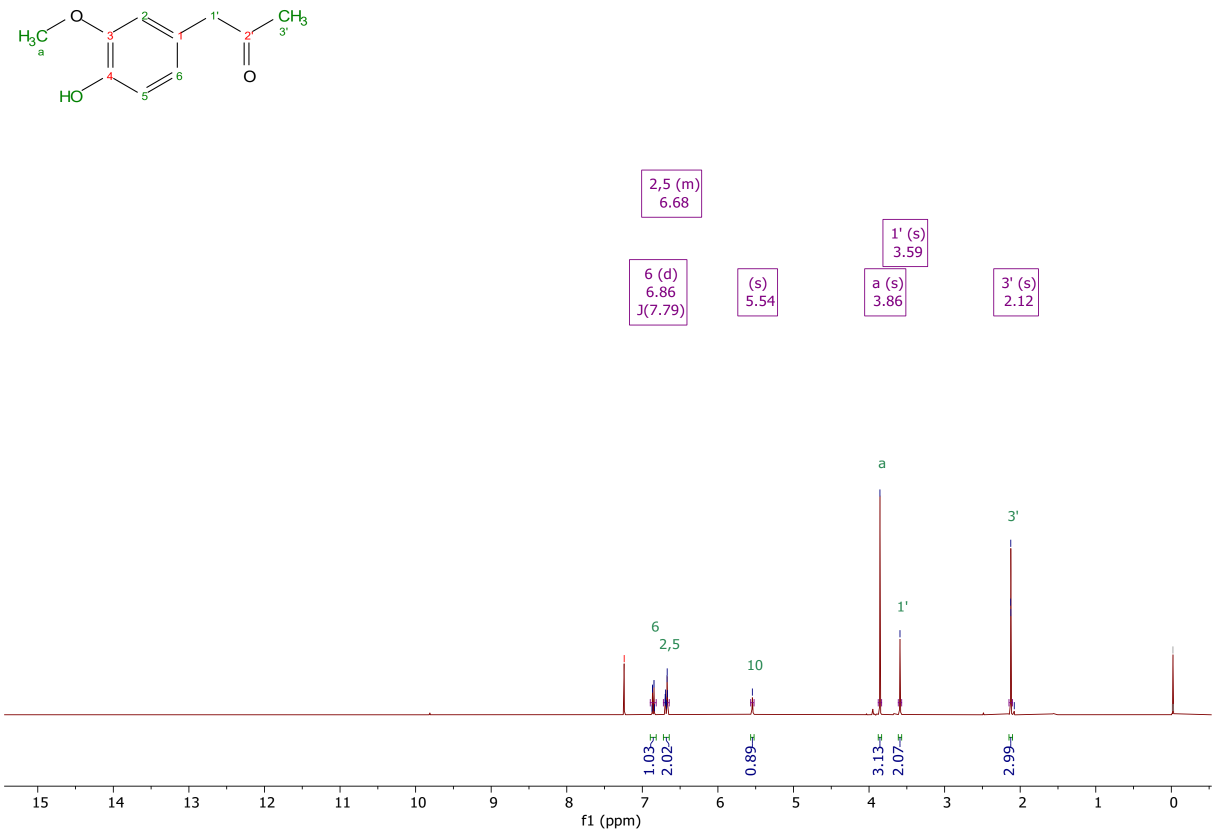
H3C a O HO 3 4 2 1 6 5 1' 2' CH3 3' O
2,5 (m) 6.68
1' (s) 3.59
6 (d) 6.86 J(7.79)
(s) 5.54
a (s) 3.86
3' (s) 2.12
a
3'
1'
6
2,5
10
1.03 2.02 0.89 3.13 2.07 2.99
15 14 13 12 11 10 9 8 7 6 5 4 3 2 1 0
f1 (ppm)

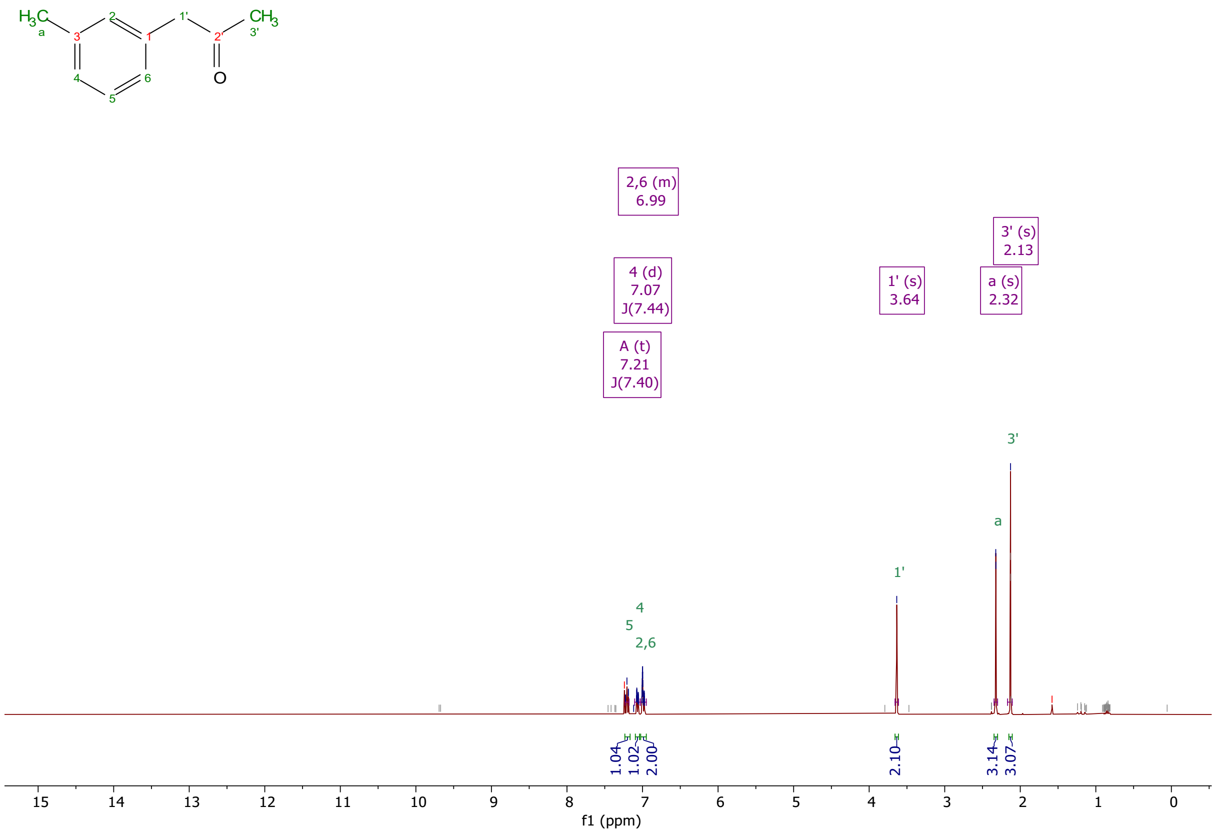
H3C
a
1'
2'
CH3
3'
O
2,6 (m)
6.99
4 (d)
7.07
J(7.44)
A (t)
7.21
J(7.40)
1' (s)
3.64
a (s)
2.32
3' (s)
2.13
1.04
1.02
2.00
2.10
3.14
3.07
f1 (ppm)

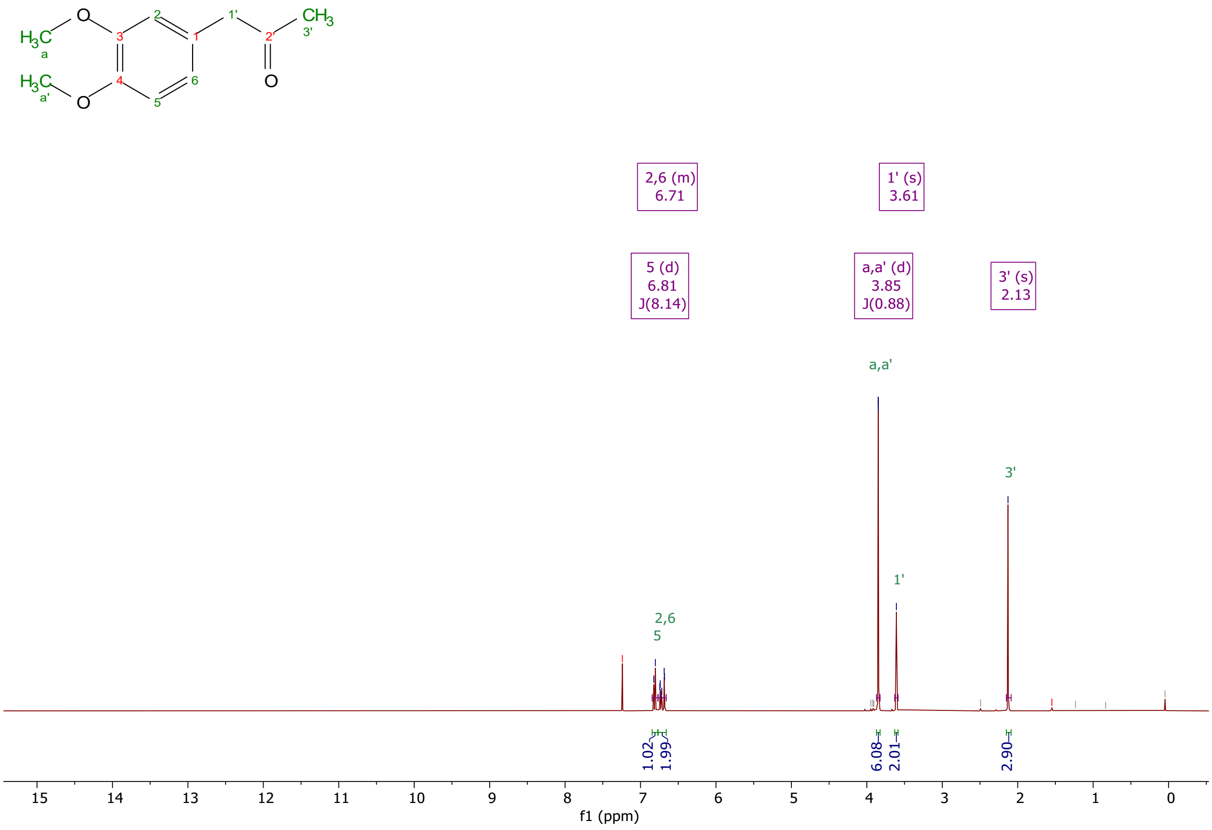
H3C
a
H3C
a'
O
O
1'
2'
CH3
3'
O
2,6 (m)
6.71
5 (d)
6.81
J(8.14)
1' (s)
3.61
a,a' (d)
3.85
J(0.88)
3' (s)
2.13
1.02
1.99
6.08
2.01
2.90
f1 (ppm)

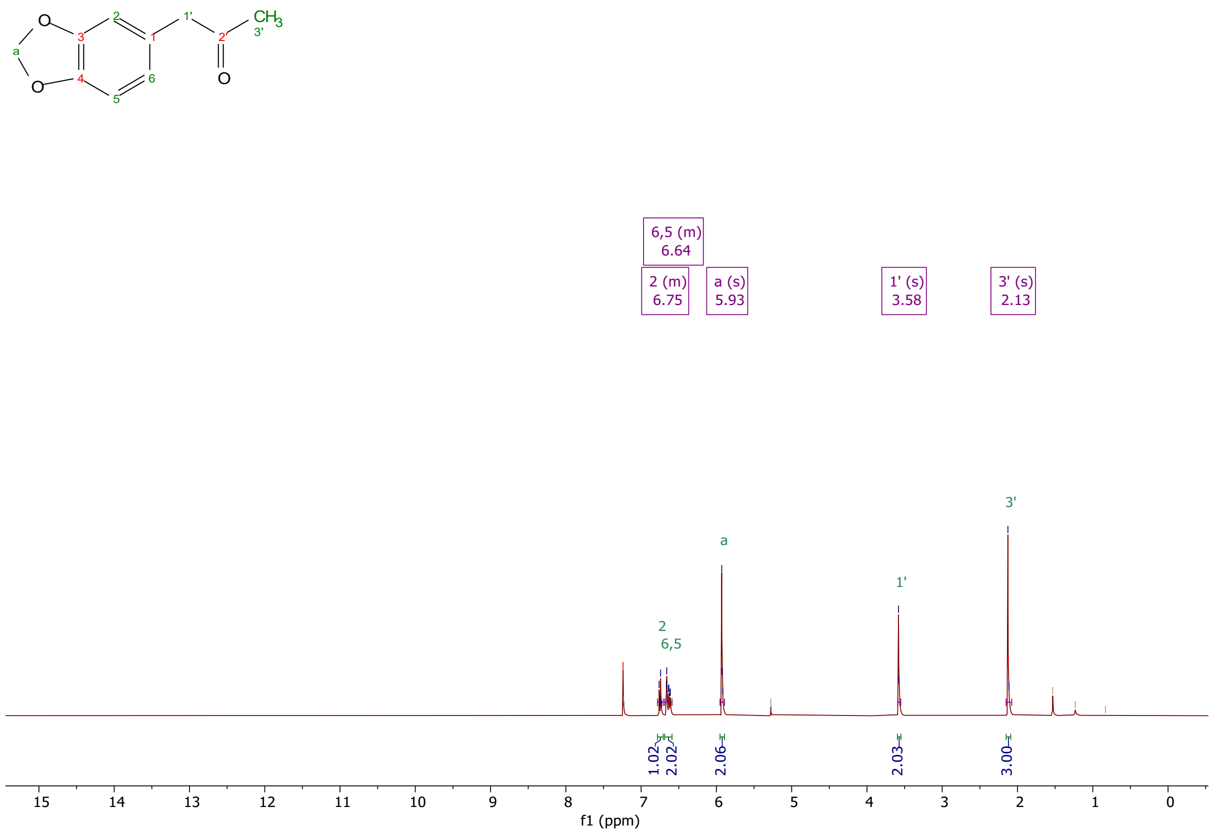
6,5 (m)
6.64
2 (m)
6.75
a (s)
5.93
1' (s)
3.58
3' (s)
2.13
1.02
2.02
2.06
2.03
3.00
f1 (ppm)

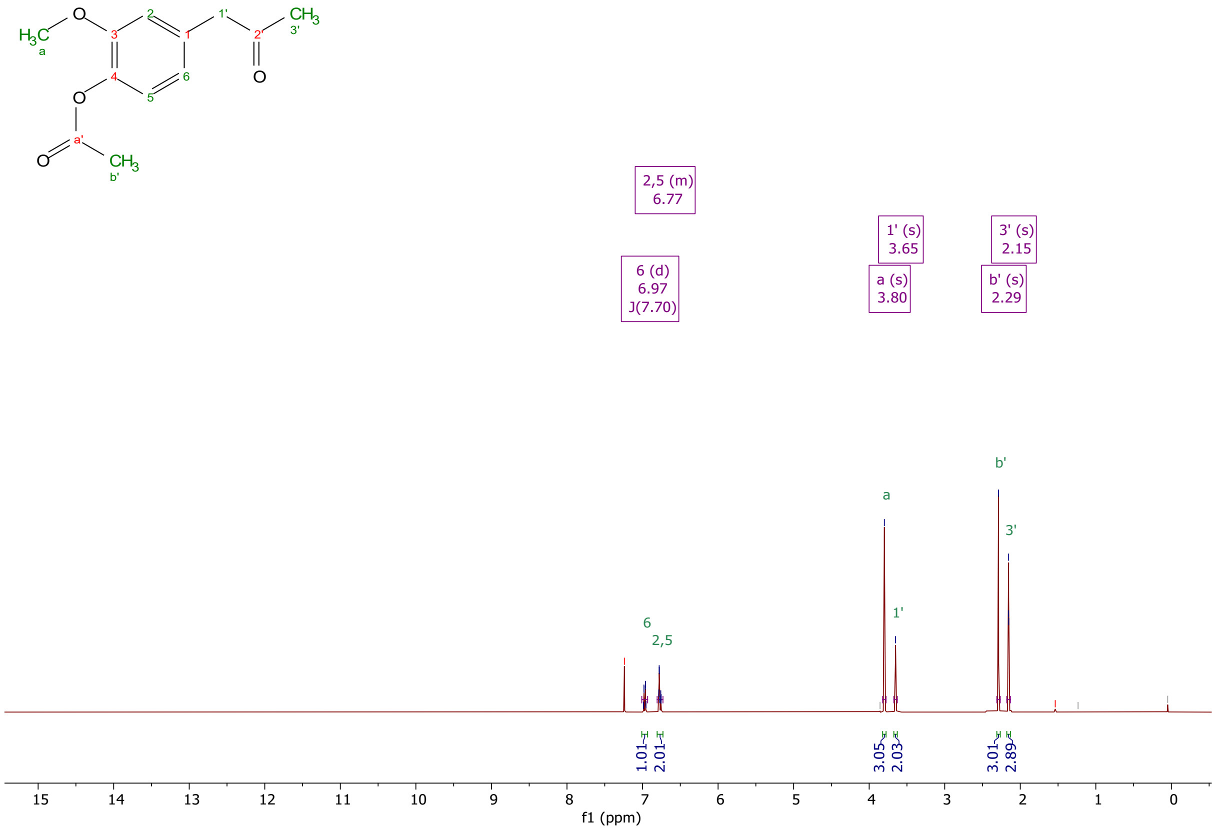
2,5 (m)
6.77
1' (s)
3.65
3' (s)
2.15
6 (d)
6.97
J(7.70)
a (s)
3.80
b' (s)
2.29
1.01
2.01
3.05
2.03
3.01
2.89
f1 (ppm)

8. Gene Sequence

wt. TmCHMO

ATGAGCACCACCCAGACCCCGGACCTGGATGCGATCGTGATTGGCGCGGGCTTCGGCGGTATCTACATGCTGC
ACAAACTGCGTAACGACCTGGGTCTGAGCGTGCGTGTTTTTGAGAAGGGTGGCGGTGTTGGCGGTACCTGGT
ACTGGAACAAGTATCCGGGCGCGAAAAGCGATACCGAAGGTTTTGTGTACCGTTATAGCTTCGACAAAGAGCT
GCTGCGTGAATACGATTGGACCACCCGTTATCTGGACCAGCCGGATGTTCTGGCGTACCTGGAGCACGTGGTT
GAACGTTATGACCTGGCGCGTGATATCCAACTGAACACCGAGGTGACCGACGCGATTTTTGATGAGGAAACCG
AGCTGTGGCGTGTTACCACCGCGGGCGGTGAAACCCTGACCGCGCGTTTCCTGGTGACCGCGCTGGGTCTGCT
GAGCCGTAGCAACATCCCGGACATTCCGGGCCGTGATAGCTTCGCGGGTCGTCTGGTTCACACCAACGCGTGG
CCGGAAGACCTGGATATCACCGGCAAGCGTGTGGGTGTTATTGGCACCGGTAGCACCGGTACCCAGTTTATCG
TGGCGGCGGCGAAAATGGCGGAGCAACTGACCGTGTTCCAGCGTACCCCGCAATACTGCGTTCCGAGCGGCA
ACGGTCCGATGGACCCGGATGAAGTTGCGCGTATCAAGCAGAACTTTGACAGCATTTGGGATCAAGTGCGTA
GCAGCACCGTTGCGTTCGGCTTTGAGGAAAGCACCGTGGAGGCGATGAGCGTTAGCGAGAGCGAACGTCAGC
GTGTGTTCCAGCAAGCGTGGGACAAAGGCAACGGTTTCCGTTTTATGTTCGGTACCTTTTGCGATATCGCGACC
AACCCGGAGGCGAACGCGGCGGCGGCGGCGTTCATTCGTAGCAAGATCGCGGAGATTGTGAAAGACCCGGA
AACCGCGCGTAAGCTGACCCCGACCGATCTGTACGCGAAACGTCCGCTGTGCAACGAGGGTTACTATGAAACC
TATAACCGTGACAACGTGAGCCTGGTTAGCCTGAAGGAAACCCCGATCGAGGAAATTGTGCCGCAAGGCGTT
CGTACCAGCGACGGTGTGGTTCACGAACTGGATGTGCTGGTTTTTGCGACCGGCTTCGACGCGGTTGATGGTA
ACTACCGTGCGATGAACCTGCGTGGCCGTGATGGTCGTCACATCAACGAGCACTGGACCGAAGGCCCGACCA
GCTATCTGGGCGTGACCAAGGCGGGTTTTCCGAACATGTTCATGATCCTGGGCCCGAACGGTCCGTTTACCAA
CCTGCCGCCGAGCATTGAGGCGCAGGTTGAATGGATCAGCGACCTGATTGATAAAGCGACCCGTGAGGGTCT
GACCACCGTGGAACCGACCGCGGATGCGGAGCGTGAATGGACCGAAACCTGCGCGGAAATTGCGAACATGA
CCCTGTTTCCGAAGGCGGATAGCTGGATCTTCGGCGCGAACATTCCGGGTAAACGTCACGCGGTTATGTTCTA
CCTGGGCGGTCTGGGTAACTATCGTCGTCAACTGGCGGACGTGGCGGACGGCGGCTATCGTGGTTTCCAACTG
CGTGGTGAACGTGCGCAAGCGGTGGCGTAA

wt. PAMO

ATGGCCGGGCAGACGACTGTCGACTCTCGCCGACAACCACCAGAGGAAGTGGACGTCCTGGTCGTGGGAGCC
GGCTTCTCCGGCCTGTACGCCCTCTACCGTCTGCGGGAACTCGGGCGTAGCGTGCACGTCATCGAAACCGCTG
GCGACGTGGGCGGTGTGTGGTACTGGAACCGCTACCCGGGGGCGCGGTGCGACATCGAGAGCATCGAGTAC
TGCTACTCGTTCTCCGAGGAGGTCCTCCAAGAGTGGAACTGGACTGAGCGGTACGCTTCCCAGCCCGAGATCC
TGCGCTACATCAACTTCGTCGCCGACAAATTCGACCTGCGGAGCGGAATCACCTTCCACACCACGGTGACCGC
GGCAGCCTTCGACGAGGCCACCAACACGTGGACGGTCGACACCAACCACGGCGACCGGATCCGCGCCCGCTA
CCTCATCATGGCCAGCGGCCAGCTCTCCGTCCCGCAGCTCCCCAACTTCCCAGGGCTCAAAGACTTCGCCGGCA
ACCTCTACCACACCGGGAACTGGCCGCACGAGCCCGTGGACTTCTCCGGGCAGCGGGTGGGCGTGATCGGCA
CCGGGTCGTCCGGAATCCAGGTGAGCCCACAGATCGCGAAACAGGCTGCGGAACTGTTCGTGTTCCAGCGCA
CCCCCCACTTTGCCGTCCCCGCCCGCAACGCGCCGCTCGACCCGGAGTTCCTCGCCGACCTGAAGAAGCGCTAC
GCCGAATTCCGGGAAGAGTCCCGCAACACTCCCGGAGGAACCCACCGCTACCAGGGGCCGAAGTCCGCGCTG
GAAGTCAGCGACGAAGAGCTGGTGGAAACCCTGGAACGCTACTGGCAGGAGGGCGGCCCCGACATTCTGGC
CGCCTACCGCGACATCCTGCGCGACCGGGACGCCAACGAACGGGTCGCTGAATTCATCCGCAACAAGATCCGC
AACACCGTGCGCGACCCGGAGGTGGCGGAACGCCTGGTCCCCAAGGGCTACCCGTTCGGCACCAAGCGCCTC
ATCCTGGAAATCGACTACTACGAAATGTTCAACCGGGACAACGTGCACCTGGTCGACACGTTGTCTGCGCCGA
TCGAGACGATCACGCCGCGCGGGGTGCGCACCTCGGAACGGGAGTACGAACTCGACTCCCTGGTGCTGGCGA
CCGGGTTCGACGCGCTGACCGGGGCGCTGTTCAAGATCGACATCCGCGGTGTGGGCAACGTCGCTTTGAAGG
AGAAGTGGGCCGCAGGGCCACGCACCTACCTGGGACTGTCCACCGCCGGGTTCCCCAACCTGTTCTTCATCGC
AGGCCCGGGCAGCCCGTCTGCGCTCAGCAACATGCTGGTCTCTATCGAACAGCACGTGGAATGGGTGACCGA
CCACATCGCCTACATGTTCAAGAACGGCCTCACCCGATCCGAGGCAGTCCTGGAGAAAGAAGACGAGTGGGT
GGAGCACGTCAACGAGATCGCCGACGAAACCCTCTACCCCATGACCGCCTCGTGGTACACGGGCGCCAACGTC
CCCGGTAAACCCCGGGTGTTCATGCTCTACGTCGGCGGCTTCCACCGCTACCGGCAGATCTGCGACGAGGTGG
CTGCCAAGGGCTACGAAGGTTTCGTCCTCACCTAG

wt. AlkJ

ATGTACGACTATATAATCGTTGGTGCTGGATCTGCAGGATGTGTGCTTGCTAATCGTCTTTCGGCCGACCCCTC
TAAAAGAGTTTGTTTACTTGAAGCTGGGCCGCGAGATACGAATCCGCTAATTCATATGCCGTTAGGTATTGCTT
TGCTTTCAAATAGTAAAAAGTTGAATTGGGCTTTTCAAACTGCGCCACAGCAAAATCTCAACGGCCGGAGCCTT
TTCTGGCCACGAGGAAAAACGTTAGGTGGTTCAAGCTCAATCAACGCAATGGTCTATATCCGAGGGCATGAAG
ACGATTACCACGCATGGGAGCAGGCGGCCGGCCGCTACTGGGGTTGGTACCGGGCTCTTGAGTTGTTCAAAA
GGCTTGAATGCAACCAGCGATTCGATAAGTCCGAGCACCATGGGGTTGACGGAGAATTAGCTGTTAGTGATTT
AAAATATATCAATCCGCTTAGCAAAGCATTCGTGCAAGCCGGCATGGAGGCCAATATTAATTTCAACGGAGAT
TTCAACGGCGAGTACCAGGACGGCGTAGGGTTCTATCAAGTAACCCAAAAAAATGGACAACGCTGGAGCTCG
GCGCGTGCATTCTTGCACGGTGTACTTTCCAGACCAAATCTAGACATCATTACTGATGCGCATGCATCAAAAAT
TCTTTTTGAAGACCGTAAGGCGGTTGGTGTTTCTTATATAAAGAAAAATATGCACCATCAAGTCAAGACAACGA
GTGGTGGTGAAGTACTTCTTAGTCTTGGCGCAGTCGGCACGCCTCACCTTCTAATGCTTTCTGGTGTTGGGGCT
GCAGCCGAGCTTAAGGAACATGGTGTTTCTCTAGTCCATGATCTTCCTGAGGTGGGGAAAAATCTTCAAGATC
ATTTGGACATCACATTGATGTGCGCAGCAAATTCGAGAGAGCCGATAGGTGTTGCTCTTTCTTTCATCCCTCGT
GGTGTCTCGGGTTTGTTTTCATATGTGTTTAAGCGCGAGGGGTTTCTCACTAGTAACGTGGCAGAGTCGGGTG
GTTTTGTAAAAAGTTCTCCTGATCGTGATCGGCCCAATTTGCAGTTTCATTTCCTTCCAACTTATCTTAAAGATCA
CGGTCGAAAAATAGCGGGTGGTTATGGTTATACGCTACATATATGTGATCTTTTGCCTAAGAGCCGAGGCAGA
ATTGGCCTAAAAAGCGCCAATCCATTACAGCCGCCTTTAATTGACCCGAACTATCTTAGCGATCATGAAGATAT
TAAAACCATGATTGCGGGTATTAAGATAGGGCGCGCTATTTTGCAGGCCCCATCGATGGCGAAGCATTTTAAG
CATGAAGTAGTACCGGGCCAGGCTGTTAAAACTGATGATGAAATAATCGAAGATATTCGTAGGCGAGCTGAG
ACTATATACCATCCGGTAGGTACTTGTAGGATGGGTAAAGATCCAGCGTCAGTTGTTGATCCGTGCCTGAAGA
TCCGTGGGTTGGCAAATATTAGAGTCGTTGATGCGTCAATTATGCCGCACTTGGTCGCGGGTAACACAAACGC
TCCAACTATTATGATTGCAGAAAATGCGGCAGAAATAATTATGCGGAATCTTGATGTGGAAGCATTAGAGGCT
AGCGCTGAGTTTGCTCGCGAGGGTGCAGAGCTAGAGTTGGCCATGATAGCTGTCTGCATGTAA

wt. PfeI

ATGAGCACATTTGTTGCAAAAGACGGTACCCAGATCTATTTCAAGGACTGGGGCAGCGGTAAACCGGTGTTGT
TCAGCCACGGTTGGCTACTGGATGCCGACATGTGGGAATACCAGATGGAGTACCTCAGCAGCCGCGGCTATC
GCACCATCGCCTTTGACCGCCGCGGCTTTGGCCGCTCGGACCAACCCTGGACCGGCAACGACTACGACACCTT
CGCCGACGACATCGCCCAGTTGATCGAACACCTGGACCTCAAGGAGGTGACCCTGGTGGGCTTCTCCATGGGC
GGCGGCGATGTGGCCCGCTACATCGCCCGCCACGGCAGCGCACGGGTGGCCGGCCTGGTGCTGCTGGGCGCC
GTCACCCCGCTGTTCGGCCAGAAGCCCGACTATCCGCAGGGTGTCCCGCTCGATGTGTTCGCAAGGTTCAAGA
CTGAGCTGCTGAAGGATCGCGCGCAGTTCATCAGCGATTTCAACGCACCGTTCTATGGCATCAACAAGGGCCA
GGTCGTCTCCCAAGGCGTGCAGACCCAGACCCTGCAAATCGCCCTGCTGGCCTCGCTCAAGGCCACGGTGGAT
TGCGTCACCGCGTTCGCCGAAACCGACTTCCGCCCGGACATGGCCAAGATCGACGTACCCACCCTGGTGATCC
ATGGCGATGGCGACCAGATCGTGCCGTTCGAGACCACCGGCAAAGTGGCGGCGGAGTTGATCAAGGGCGCC
GAACTGAAGGTGTACAAGGACGCGCCCCACGGGTTCGCGGTGACCCACGCCCAGCAGTTGAACGAAGACCTG
TTGGCGTTCTTGAAACGCTGA

wt. ADO

TTAAGTATAAGAAGGAGATATACATATGCATCATCACCATCACCATGCTCACATCCACGACCTGGCGCCGGAA
GTAAGCAACTACTCTTCTGGTCGCCTGACCCCGCCGACCCCAGTTAGGTTCCCGCGCACCCCAGTGTTCGCATC
TATGAACAAACCGTGCCGCTTCGAAGGTGACGTTTTCGACCTGGAAGTTTCTGGTGCTATCCCGCCGGACATCG
ACGGTACCTTCTTCCGCGTTCAGCCGGACCACCGCTTCCCGCCGCTGTTCGAAGACGACATCCACTTCAACGGT
GACGGTTCTGTTACCGCTATCCGCATCTCTGGTGGTCACGCTGACCTGCGCCAGCGCTACGTTCGCACCGAACG
CTACCTGCTGGAAACCCGCGCTCGCCGCTCTCTGTTCGGTCGCTACCGCAACCCGTGGACCGACAACGAATCTG
TTCGCGGTGTTATCCGCACCGCTTCTAACACCAACGTTGTTTTCTGGCGCGGTGCTCTGCTGGCTATGAAAGAA
GACGGTCCGCCGTTCGCTATGGACCCGGTTACCCTGGAAACCCTGGGTCGCTACGACTTCGAAGGTCAGATCC
TGTCTCCGACCTTCACCGCTCACCCGAAAATCGACCCGGACACCGGTGAAATGGTTTGCTTCGCTTACGAAGCT
GGTGGTGACGGTTCTGACTGCTCTGTTGACGTTGCTGTTTGGACCGTTGACGCTGACGGTAAAAAAGTTGAAG

AATGCTGGTACAAAGCTCCGTTCGCTGGTATGATCCACGACTGCGGTATCACCAAAAACTGGGTTGTTCTGCCG
CTGACCCCGATCAAAATGGACCTGGAACGCATGAAACGCGGTGGTAACAAATTCGCTTGGGACCCGTCTGAAG
ACCAGTGGTACGGTGTTGTTCCGCGCCGCGGTGCTAAATCTGACGACATCATCTGGTTCCGCGCTGACAACGG
CTTCCACGGTCACGTTGCTGGTTGCTACGAACTGCCGTCTGGTGAAATCGTTTTCGACCTGACAGTTGCGGACG
GCAACGTCTTCTTCTTCTTCCCGCCGGACGACAACATCACCCCGCCGGCTGACGGTGTTGCTAAACGCAACCGC
CTGTCTTCTCCGACCGTTCGCTGGATCTTCGACCCGAAAGCTAAAAAATCTGCTATCCGCACCGAAGCTGCTGG
TGACGCTGACATCTGGGTTGCTGACGAACGCGTTAAACCGGCTCTGACCTGGCCGACCAACGGTGAATTCTCT
CGCATCGACGACCGCTACGTTACCAAACCGTACCGCCACTTCTGGCAGGCTGTTGTTGACCCGACCCGCCCGTA
CGACTTCGAAAAATGCGGTCCGCCGGCTGGTGGTCTGTTCAACTGCCTGGGTCACTACACCTGGTCTGACCAG
AACTACCACCACGGTCACAACACCGGTGACCCGTCTGGTGACGGTCGCTCTAACGGTTCTGCTGAAGAAGCTA
CCGCTGGTAAATTCGGCCTGCAGGACGTATACTTCGCCGGTCCGACCATGACCTTCCAGGAACCGACCTTCATC
CCGCGCCAGGGTGCTGCTGAAGGTGAAGGTTACCTGATCGCTCTGCTGAACCACCTGGACGAACTGCGCAAC
GACGTTGTTATCTTCGAAGCTCGCAACCTGGGTAAAGGTCCGCTGGCTGTTATCCACCTGCCGCTGAAACTGAA
ACTGGGTCTGCACGGTAACTGGGTTGACTCTCGCGAAATCGAAGCTTGGCGCCGCCGCCGCGCTGAAAACGG
TGACGTTGGTCCGCTGCGCGTTGCTAAAGAACCGCTGCCGTGGCAGAAAAAATTCGCTGCTGCTGCTCAGAAC
GGTTCTAACGGTGTTTAA

9. Literature

(1) Krebsfänger, N.; Zocher, F.; Altenbuchner, J.; Bornscheuer, U. T., Characterization and enantioselectivity of a recombinant esterase from *Pseudomonas fluorescens*. *Enz. Microb. Technol.* **1998,** *22*, 641-646.

(2) González-Martínez, D.; Gotor, V.; Gotor-Fernández, V., Stereoselective synthesis of 1-arylpropan-2-amines from allylbenzenes through a Wacker-Tsuji oxidation-biotransamination sequential process. *Adv. Synt. Catal.* **2019,** 361, 2582.

(3) Sang, D.; Tian, J.; Tu, X.; He, Z.; Yao, M., Cleavage of catechol monoalkyl ethers by aluminum triiodide–dimethyl sulfoxide. *Synthesi* **2019,** *51*, 704-712.
